# Collision-induced activation: Towards industrially scalable approach to graphite nanoplatelets functionalization for superior polymer nanocomposites

**DOI:** 10.1038/s41598-017-03890-8

**Published:** 2017-06-15

**Authors:** Omid Zabihi, Mojtaba Ahmadi, Tahereh Abdollahi, Saeid Nikafshar, Minoo Naebe

**Affiliations:** 1 Deakin University, Carbon Nexus, Institute for Frontier Materials, Geelong, Australia; 2 Department of Chemical Engineering, Isfahan University of Technology, Isfahan, 84156/83111 Iran; 3 Department of Physical Chemistry, University of Mazandaran, Babolsar, Iran; 4 Department of Applied Chemistry, Faculty of Chemistry, University of Tabriz, Tabriz, Iran

## Abstract

Scale-up manufacturing of engineered graphene-like nanomaterials to deliver the industry needs for development of high-performance polymer nanocomposites still remains a challenge. Herein, we introduce a quick and cost-effective approach to scalable production of functionalized graphite nanoplatelets using *“kitchen blender”* approach and Diels-Alder chemistry. We have shown that, in a solvent-free process and through a cycloaddition mechanism, maleic anhydride can be grafted onto the edge-localized electron rich active sites of graphite nanoplatelets (GNP) resulting from high collision force, called “*graphite collision-induced activation*”. The mechanical impact was modelled by applying the point charge method using density functional theory (DFT). The functionalization of GNP with maleic anhydride (*m*-GNP) was characterized using various spectroscopy techniques. In the next step, we used a recyclable process to convert *m*-GNP to the highly-reactive GNP (*f*-GNP) which exhibits a strong affinity towards the epoxy polymer matrix. It was found that at a low content of *f*-GNP e.g., 0.5 wt%, significant enhancements of ~54% and ~65% in tensile and flexural strengths of epoxy nanocomposite can be achieved, respectively. It is believed that this new protocol for functionalization of graphene nanomaterials will pave the way for relatively simple industrial scale fabrication of high performance graphene based nanocomposites.

## Introduction

Among different carbon nanostructures, graphene, a single layer of sp^2^ carbon atoms arranged in a honeycomb structure, has received numerous attentions due to its high surface area and prominent mechanical, thermal and several other unique properties, since its discovery in 2004^1^. The mechanical properties of an ideal single layer graphene such as Young’s modulus and strength are ca. 1 TPa and ca. 130 GPa, respectively, making graphene one of the strongest materials. Single and few layered graphene can mainly be produced by two different approaches; bottom-up and top-down techniques^2^. Bottom-up methods, such as epitaxial growth and chemical vapor deposition, can yield high quality graphene with minor defects; however, these methods are costly and hence not suitable for scaling up for the purpose of industrial-scale production^3^. Compared with bottom-up methods, top-down techniques such as oxidation–reduction process (e.g. Staudenmaier, Brodie, and Hummers’ based methods), and direct micro-mechanical exfoliation of highly ordered pyrolytic graphene in the liquid phase^4^, have the potential to produce large amounts of graphene-like nanoplatelets^5^. Nevertheless, there are some downsides when using these techniques including defects induced to the basal-plane of graphene^6^. On the other hand, sonication have been widely reported in literature, as a method for micromechanical assisted liquid phase exfoliation of graphene that could result in the large-scale production of graphite through cavitation and its wedge effect^7^. However, sonication is known to introduce harsh conditions (e.g. high local temperature, extreme pressure and rapid heating/cooling rates) which could lead to flaws on the edges and “hole-like” defects on basal planes of graphene^8, 9^. Recently, the humble “*kitchen blender*” has been utilized to achieve scalable manufacture of graphene^10^. In comparison with other techniques such as ball milling and sonication, a fully turbulent flow generated by kitchen blender possesses the combination of shear and nominal force needed for exfoliation mechanism^8^. Consequently, much higher exfoliation efficiency is obtained. Additionally, mild processing conditions and eco-friendly features of this method results in the almost defect-free and high surface-area graphene fabrication^8, 10–12^. Once effective exfoliation is achieved, surface chemistry of graphene plays a critical role as it influences the suitability of graphene for different applications. A commercially available and fairly low-cost form of graphene is graphite nanoplatelets (GNPs), constituted by few stacked sp^2^ graphene layers possessing oxygen containing functional groups. These GNPs are usually prepared by intercalating graphite either with metal ions or by acid treatment, which is then further exfoliated via thermal shocking^13, 14^.

The inclusion of these GNPs as structural reinforcement fillers is expected to improve matrix-dominated mechanical and thermal properties including strength, stiffness, and thermal stability. One of the most desirable applications of GNPs is in fabrication of high-performance epoxy nanocomposites^15, 16^. Epoxy resins are widely used in a number of different areas such as aerospace, automotive, sports materials, construction, electrical and electronic systems^17–19^. However, epoxy materials are often limited by their inherited brittleness and poor thermal properties. A simple approach to overcome this problem could be modification of the matrix using GNPs^20, 21^. Compared to other carbon based nanofillers, the larger surface area of GNPs and higher contact area between GNPs and epoxy matrix increase the interfacial interaction hence improving the stress transfer from the polymer to the nanoplatelets. Nevertheless, the ability to achieve a uniform dispersion of GNPs within the polymer matrix still remains a challenge in industrial application of graphite nanoplatelets. This is mainly due to the strong Vander Waals forces and π-π inter-planer stacking resulting in strong tendency to form GNPs agglomerates^22, 23^. Moreover, the lack of interfacial bonding between GNPs and epoxy matrix hinders the load transfer from matrix to GNPs. To this end, several methods such as ultrasonication, high-shear mechanical mixing, use of surfactants, mechanical alignment, chemical modification, and polymer chains wrapping have been utilized to achieve both the homogenous dispersions well as effective interfacial interaction. Among these methods, chemical functionalization of the GNPs is very appealing as it can provide the chemical affinity which is required to achieve uniform dispersion and interfacial bonding. The chemical functionalization of GNPs via reactive linker molecules can provide the required chemical reactions between linkers and functional groups of epoxy matrix which consequently leads to the effective load transfer to the GNPs. As a result, it is expected that the possibility of interfacial debonding between GNPs and epoxy matrix drops significantly^24, 25^. Compared with non-covalent chemical modification of graphene, the covalent functionalization is accompanied by rehybridization of sp^2^ carbon atoms to the sp^3^ configuration accomplished by the loss of electronic conjugation. The covalent chemistry assuring good bonding between the graphene and modifying agents can be obtained via four different approaches: nucleophilic substitution, electrophilic addition, condensation, and addition^26–28^. Among these methods, wet chemical functionalization of graphene such as hydrogenation, cycloaddition reactions (e.g. Aryne cycloaddition, Diels-Alder and Bingel reactions), addition of diazonium species, nitrene addition, and acylation reactions have been used to tailor the surface chemistry of graphene oxide, reduced graphene oxide, and GNPs within epoxy polymers in order to modify their dispersion levels and improved interfacial interactions^29–33^.

Most of these approaches require relatively demanding reaction conditions and/or several synthetic steps which are prerequisite to functionalize the graphene based nanomaterials. This mostly and significantly interfere with industrial requirements for large-scale production. Recently, development of functionalised graphene by Diels-Alder reactions has received great attention mainly due to the mild conditions required in this approach. Diels-Alder reactions are well-known to occur between a conjugated diene and a dieneophile, in which graphene usually acts as a diene^34–36^. Some *“electron poor”* alkenes compounds such as maleic anhydride, maleimid^35, 37^, tetracyanoethylene^35, 38^, and tetracyanoethylene oxide^39^ have been reported to be covalently, thermally, and reversibly attached onto graphene and graphite as dieneophile species by Diels-Alder cycloaddition. Sarkar *et al*.^35^ performed the Diels-Alder reaction of maleic anhydride onto the various graphite types, however, a large quantity of organic solvent is required due to low dispersion stability of graphite in organic solvents, and there is no report on yield obtained through this process. Moreover, a fairly costly and time-consuming ball milling process of graphite led to edge-selected functionalization of graphene in the presence of maleic anhydride and maleimid using Diels-Alder cycloaddition mechanism^37^.

For the first time, in this work, we introduce a feasible, scalable, and solvent-free method for functionalization of commercially available GNPs. This is achieved by combining the “*kitchen blender*” approach and Diels-Alder chemistry, in which maleic anhydride was simultaneously used as stabilizing agent in “*kitchen blender*” approach and dieneophile in Diels-Alder cycloaddition reaction. This method was utilized in production of tailored GNPs to be purposefully incorporated into epoxy polymer matrix as a potential industrial application. The thermo-physical and interfacial properties of the epoxy nanocomposites were evaluated to demonstrate the effectiveness of our proposed functionalization approach.

## Results and Discussions

### Nanoplatelets characterizations

**Figure 1:**
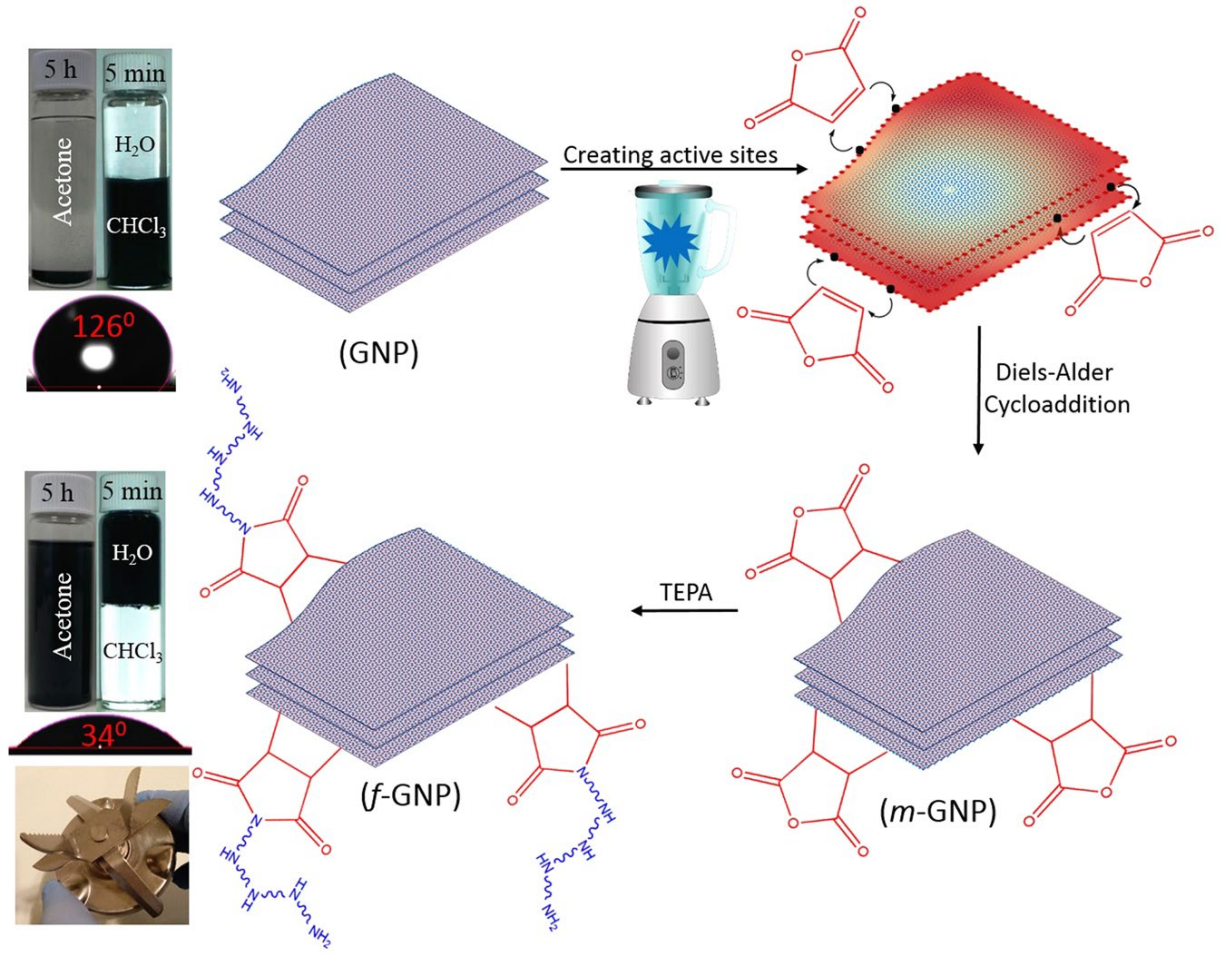
Kitchen blender assisted Diels-Alder based chemical synthesis of amino-functionalized graphene; and its solubility profiles and water contact angle.

Figure 1 displays the chemical route designed for functionalization of GNPs using a kitchen blender. It has been discovered very recently that electrons of the graphite structure have a honey-like viscose flow through its plane, forming by intrinsically electron-electron collisions^40^. Since the graphite structures consist of un-pair π electrons uniformly resonated on the graphene plane^40, 41^, it is hypothesized that high-energy collisions could induce an electron flow towards the edge. This means that a temporary local negative charge could be formed for a short while preferably at the plane edge when collision of nanopatlates with blades occur, leading to the activation of nanopatlates which we call “graphene collision-induced activation”. According to the proposed mechanism, maleic anhydride (MA), acting as a dienophile, covalently reacts with these electron-rich active sites. In other words, MA stabilizes these active sites through its LUMO orbitals by forming sigma bonds with highly-activated HOMO orbitals of GNP edges. The proposed mechanism and its hypotheses were investigated and confirmed by DFT computational and experimental methods.

### Theoretical modelling

The point charge method was used, for the first time, in order to model a perturbation for the distribution of unpaired π electrons and flow of electric charge in the nanoplatelets as a result of mechanical collisions. Under a mechanical impact the graphite edges are temporarily negatively charged due to the polarization of delocalized π electrons. Applying the point charge in the center of nanoplatelets can polarize the π electrons, and therefore, the point charge can help to model the mechanical impact. In fact, the point charge causes the electron-electron repulsion in the same way as the mechanical impact does. The point charge method is a new theoretical approach, recently proposed by Baturin *et al*.^42^ for calculation of the Cherenkov radiation phenomenon. The Cherenkov radiation is an electromagnetic radiation emitted when a charged particle, such as an electron, passes through a dielectric medium at a speed greater than the phase velocity of light in that medium^43^. In this work, in order to model an electron-electron collision, we put a negative point charge in the center of the nanoplatelets. The electric field is created around this point charge making a perturbation in the electron distribution in the nanoplatelets. The electric field of a point charge can be obtained by Gauss law. Considering a Gaussian surface in the form of a sphere with radius (*r*), the electric field has the same magnitude at every surface with the same *r* and is directed outward. The electric field (*E*_field_) at radius *r* is then given by the following equation^44^:

1 $${E}_{{\rm{field}}}=\frac{Q}{4\pi {\varepsilon }_{0}{r}^{2}}$$

where *Q* and ε_o_ are charge and vacuum permittivity, respectively.

**Figure 2:**
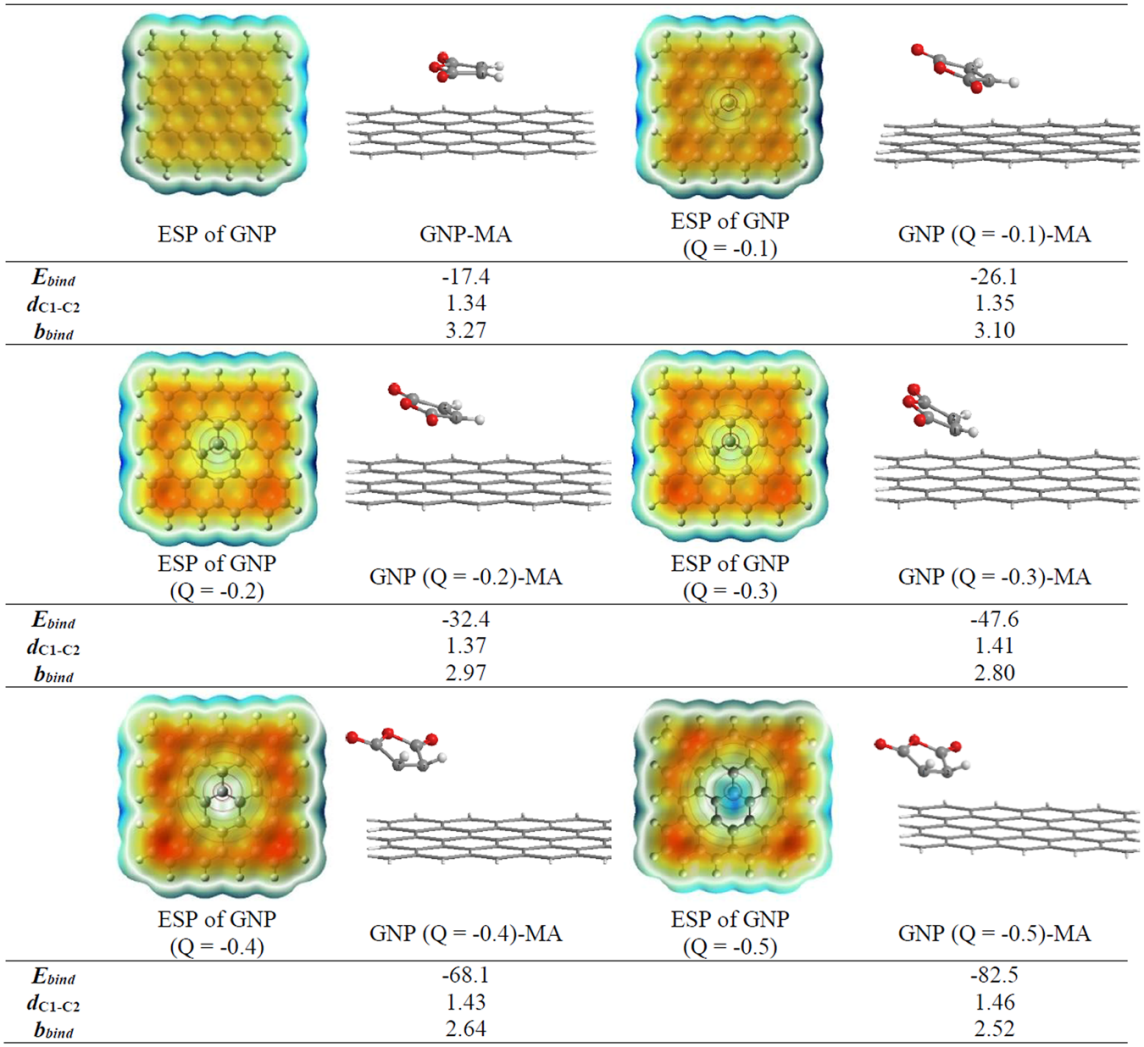
The electrostatic potential surface (ESP) generated at B97-D/6 − 31 + G* level of theory. Red and blue colors signify the regions with charge accumulation and depletion, respectively. Counterpoise corrected binding energies (***E***_***bind***_) in kcal/mol, C-C bond lengths (***d***_***C1-C2***_) and binding distances (***b***_***bind***_) in Å, for the studied GNP-maleic anhydride (MA) system.

Accordingly, the generated electric field depends on the value of the point charge (Q). Herein, we applied various point charges of −0.1, −0.2, −0.3, −0.4, and −0.5 to study the effect of applied electric field and flow of charge in the nanoplatelets on its interactions with the maleic anhydride. Figure 2 shows the charge distribution over the nanoplatelets, visualized through electrostatic potential surface (ESP) map. This map was generated at B97-D/6 − 31 + G* level, with iso-surfaces of 0.001 electrons, in au^−3^, through Gauss View package^45^. The main priority of ESP visualization is to find the reactive sites of the molecules to design an effective interaction between two structures. According to the conventional colour spectrum in ESP maps, the sites with the lowest values of electrostatic potential energy are in red (negative charge) and those with the highest values are in blue colour (positive charge), which indicate the relative abundance and absence of electrons in these regions, respectively. According to Fig. 2, with the increase of Q, which leads to the increase of the applied electric field, the electrons flow more to the outer edges of the nanopatlates. For example, in the nanopatlates structure with the point charge of −0.5, the central atoms have positive charge while the carbon atoms at the edge of nanopatlates contain negative charge. These negative charges (red colour) have been only created upon a collision and afterward the charge distribution come back to initial state with a uniform electron distribution.

The most stable structures of GNP and MA were used to study the interaction in the substrate-electrophile system. The counterpoise corrected binding energies (*E*_bind_) are presented in Fig. 2. Accordingly, the structures with the higher values of point charge are more thermodynamically stable, such that increasing the point charge from −0.1 to −0.5 results in a change in the binding energy from −26.1 to −82.5 kcal/mol. Also, the distance between the GNP and MA (*b*_bind_) decreases with increasing point charge revealing that there is a more effective adsorption. Comparison between the binding energy values of GNP-MA and GNP (Q = x)-MA complexes in Fig. 2 clearly shows that GNP (Q = x)-MA interactions are preferred over GNP-MA, which is in agreement with our obtained experimental results on the role of mechanical collisions in the activation of GNP. Considering the binding energies, it can be concluded that the adsorption of maleic anhydride on the activated GNP, i.e., GNP (Q = x), is more effective than that on the GNP. The C1-C2 bond length (1.46 Å) of MA grafted on the GNP (Q = −0.5)-MA shows an increase of 0.12 Å with respect to that of GNP-MA (1.34 Å). This is due to the more charge transfer from GNP (Q = −0.5) to C1-C2 bond of MA, compared to the GNP, which results in increasing the C1-C2 bond length indicating a change in the bond order and bond character between the carbon atoms. Also, the C1-C2 stretching frequencies in GNP (Q = −0.5)-MA and GNP-M are 1135 cm^−1^ and 1645 cm^−1^, respectively, denoting that C1-C2 in GNP (Q = x)-MA is re-hybridized when adsorption occurs and C1-C2 bonds in GNP (Q = −0.5) are more sp^3^-like than sp^2^. The C-H bonds of MA in this structure, calculated to be 1.09 Å in length, are distorted out of the initial plane of the molecule where the hydrogen atoms tilt away from the surface, as presented in Fig. 2.

### Experimental characterizations

After grafting the anhydride functional groups on the GNP, it undergone a nucleophilic attack by amine groups of TEPA leading to the formation of the imide groups. The first expected change in the performance of the nanoplatelets after functionalization is their dispersion stability in various solvents. The solubility profiles of functionalised GNPs are presented in Fig. 1. In most graphite-based applications e.g. polymer nanocomposites, the poor dispersion in organic solvents is the major shortcoming for the uniform incorporation into the polymer matrix. As it can be seen, the dispersion stability of *f*-GNP in acetone remarkably increases compared to GNP due to the presence of alkyl amine groups on the *f*-GNP. Moreover, as it can be seen from the water contact angle data, GNP has a hydrophobic inherent leading to pulling down the GNP into hydrophobic solvent (chloroform) instead of being in hydrophilic solvent (water). After chemical functionalization, the *f*-GNP was quickly pulled up into the hydrophilic solvent as a result of hydrogen bonding of water with amine groups of *f*-GNP, confirmed by its water contact angle as well.

**Figure 3:**
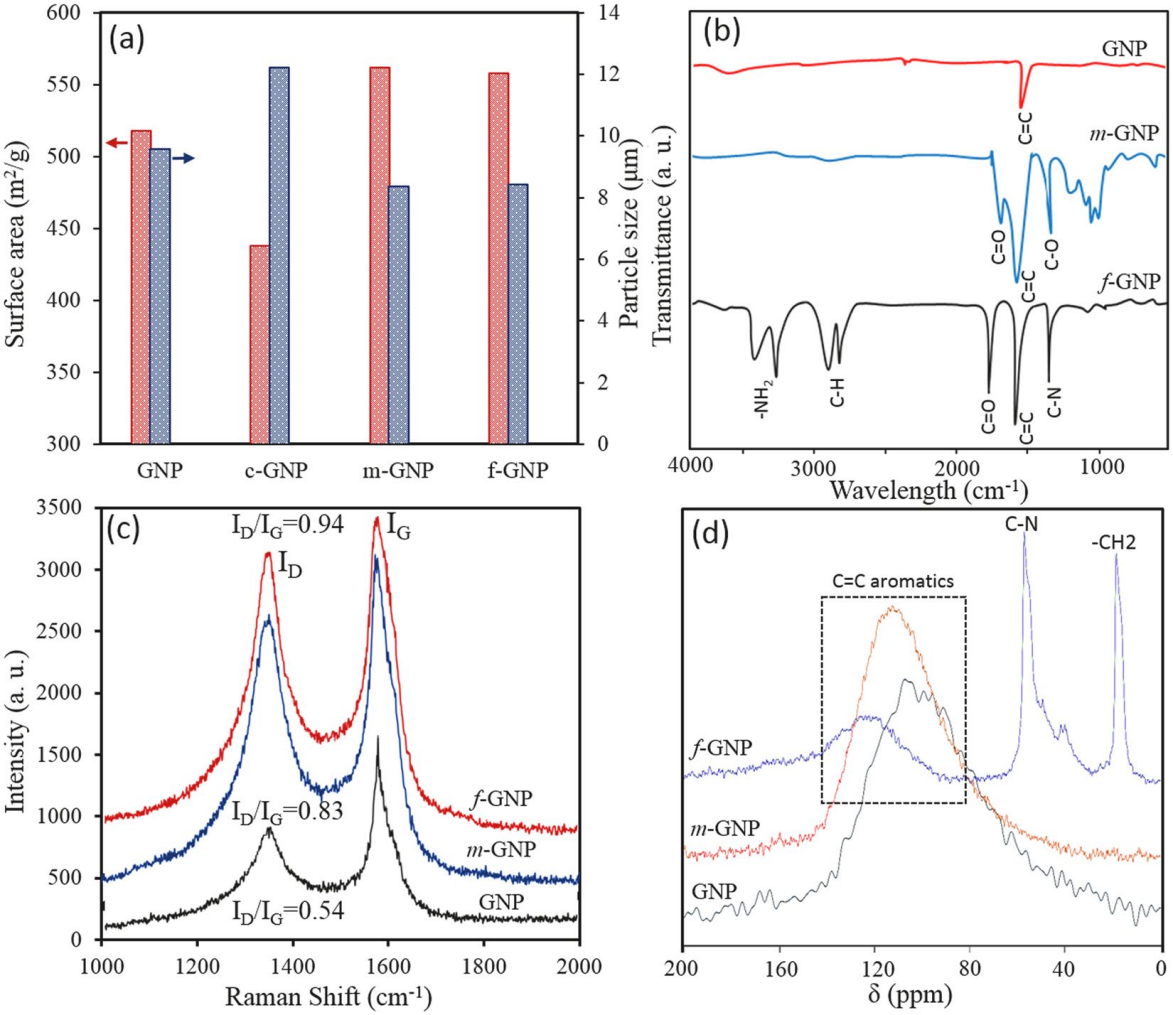
Surface area and particle size results (**a**), FTIR spectra (**b**), Raman spectra (**c**), and C^13^NMR spectra (**d**) of GNP, *m*-GNP and *f*-GNP.

**Figure 4:**
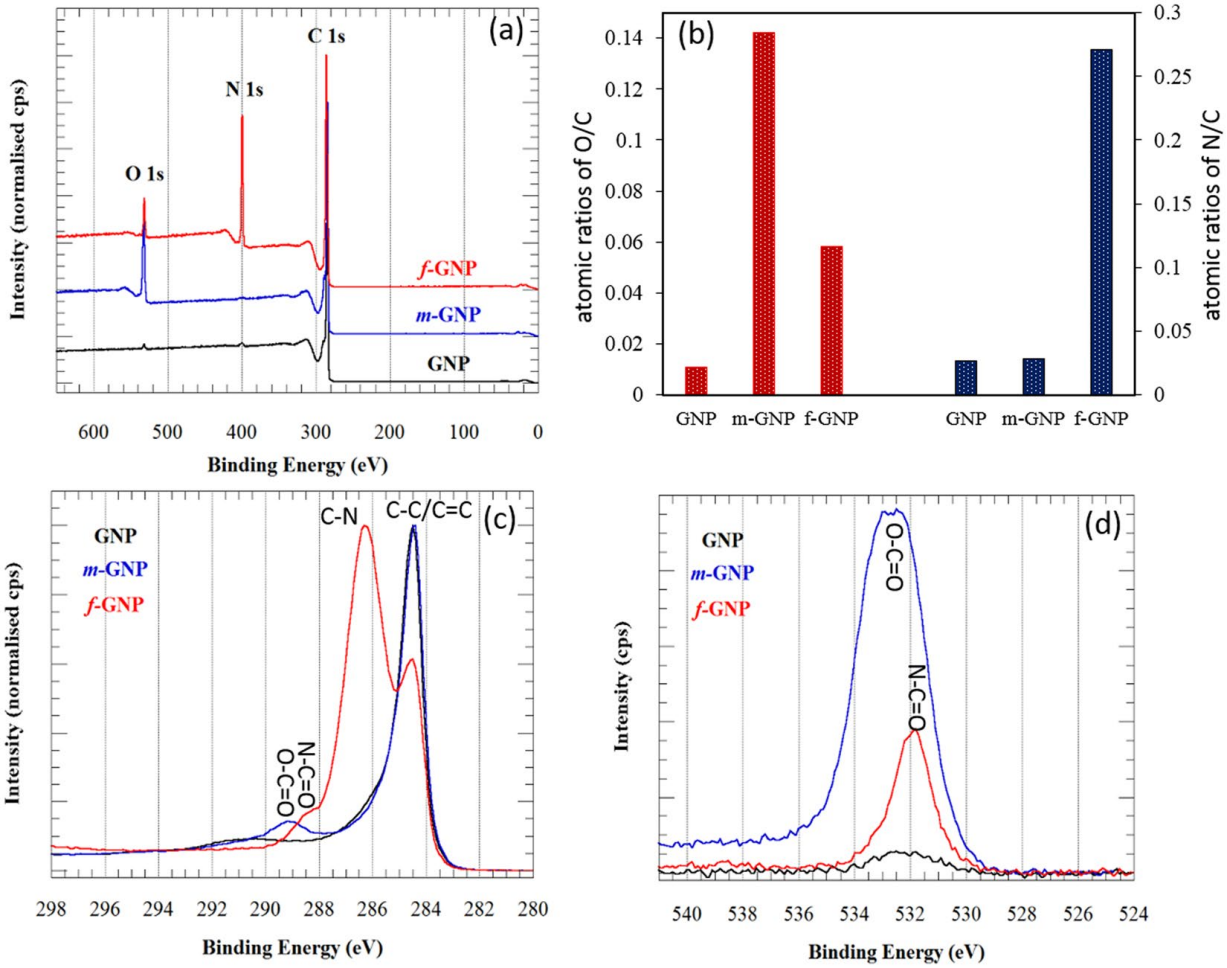
X-ray photoelectron spectra measured on the various samples; survey spectrum (**a**), O/C and N/C atomic ratios when C/C was considered to be 1 (**b**), C 1 s (**c**), and O 1 s (**d**) spectra.

It is observed that *m*-GNP and *f*-GNP are of slightly higher surface area and smaller particle size compared to pure GNP (see Fig. 3a). This shows that, in contrast to ball milling which induces a very high energy levels, only the sub-aggregation of nanoplatelets could be broken as a result of collusion with blades, resulting in smaller particle size due to the applying the high collision force followed by quick *in situ* stabilization by maleic anhydride, leading to increase in surface area. It is also hypothesised that creating electron-rich active sites without the presence of stabilizing agent of maleic anhydride (*c*-GNP) can cause re-aggregation of nanoplatelets and forming more sub-aggregation, as evidenced by a decrease of 80 m^2^/g in surface area and an increase of 2.65 µm in particles size when compared with pure GNP. The *m*-GNP and *f*-GNP have a Brunauer–Emmett–Teller (BET) surface area of 562 and 558 m^2^/g, respectively, which are mostly higher than those reported by ball-milling of graphite in the presence of other stabilizing agents^46, 47^. As seen from Fig. 3b, apart from sharp peaks of graphene skeleton of C=C bonds (ring stretching) appeared around 1585 cm^−1^ and weak shoulder-peak of hydroxyl groups at around 3600 cm^−1^, pure GNP shows a featureless Fourier transform infrared (FTIR) spectra^48^. Standard characteristics peaks related to the anhydride functional groups are observed in FTIR spectra of *m*-GNP. The peaks observed in 1229 and 1728 cm^−1^ are attributed to C-O and C=O, respectively, confirming the grafting of maleic anhydride on nanoplatelets system. After amino-functionalization-based nucleophilic attack on anhydride groups, new peaks of C-N and –NH_2_ were appeared in *f*-GNP FTIR spectra at 1374 cm^−1^ and 3324–3501 cm^−1^, respectively. The C=O peak associated to the imide groups also shifted to the higher wavelengths in comparison to the C=O of anhydride groups (see Fig. 3b). Raman analysis was applied as a useful tool to evaluate the functionalization degree of nanoplatelets. Figure 3c shows the raman spectra of the nanoplatelets, in which all samples have the characteristics of D band at 1352 cm^−1^ (the breathing mode of C-sp^2^ atoms in the rings and is related to the disorder presence within the structure) and a G band at 1572 cm^−1^ (the in-plane bond stretching motion of C-sp^2^ atoms)^49, 50^. The I_D_/I_G_ ratio of GNP is about 0.45, which typically is related to the some functional groups in pure GNP, generated during its manufacturing and air moistures absorption. Raman spectrum of *m*-GNP and *f*-GNP shows an increment in I_D_/I_G_ ratio from 0.45 to 0.83 and 0.99, respectively, in comparison with GNP, attributed to the enhancement in defect concentration due to functionalization, i.e., conversion of π-bonded C-sp^2^ carbons to C-sp^3^. Moreover, the higher I_D_/I_G_ ratio signifies a higher degree of covalent functionalization^35, 51, 52^. It can be also observed that G band of *m*-GNP and *f*-GNP at 1579 cm^−1^ became slightly sharper. As shown in Fig. 3d, crbon-13 nuclear magnetic resonance (^13^C-NMR) spectroscopy further substantiates the functionalization of GNP, in agreement with the above results. Characteristic C^13^-NMR peak of aromatic sp^2^ carbons appear normally at 110–150 ppm. In all samples, chemical shift of C=C in aromatic structures of graphite appeared at 110–130 ppm. However, anhydride groups on *m*-GNP cannot be directly detected in its spectrum which may be due to lower ratio of C=O/C=C aromatics and it shows only the peak related to the C=C. As it can be seen, the peak of C=C of GNP at 110 ppm shifted to higher chemical shifts e.g. 120 ppm for *m*-GNP and 130 ppm for *f*-GNP, which is due of the change in the chemical environment of the sp^2^ carbons as a result of withdrawing electron effect of the grafted maleic anhydrides and their further imide bonds on sp^2^ carbons, leading to be deshielded nucleuses. ^13^C-NMR of *f*-GNP reveals peaks long chain alkyl amines including C-N and –CH_2_ at 58 ppm and 18 ppm, respectively. As presented in Fig. 4a and b, it is also observed using X-ray photoelectron spectroscopy (XPS) analysis that pure GNP has a very low oxygen and nitrogen contents respect to the carbon content. While, oxygen content of *m*-GNP and nitrogen content of *f*-GNP have been significantly increased after their related functionalizations. Figure 4c demonstrates presence of O-C=O peak related to the anhydride groups in *m*-GNP. While C1s spectrum of *f*-GNP shows imide groups by presenting N-C=O and C-N peaks. These peaks also are confirmed by O1s spectrums, in which O-C=O and N-C=O are obvious in spectra of *m*-GNP and *f*-GNP, respectively. Thermogravimetry analysis (TGA) results also show significant differences of ~8.5% and ~32.5% at 600 °C in weight loss of *m*-GNP and *f*-GNP with pure GNP, respectively, confirming successfully grafting of the corresponded molecules on the nanoplatelets (see Fig. S1). These weight loss can result from both degradation of molecules on the surface and retro Diels-Alder reactions at high temperatures. Using these differences in weight loss, it is estimated that ~92 mg maleic anhydride would be totally grafted on 1 gr pure GNP during the proposed process (see supplementary information for details of calculations and assumptions).

### The potential application in epoxy nanocomposites

#### Mechanical properties

**Figure 5:**
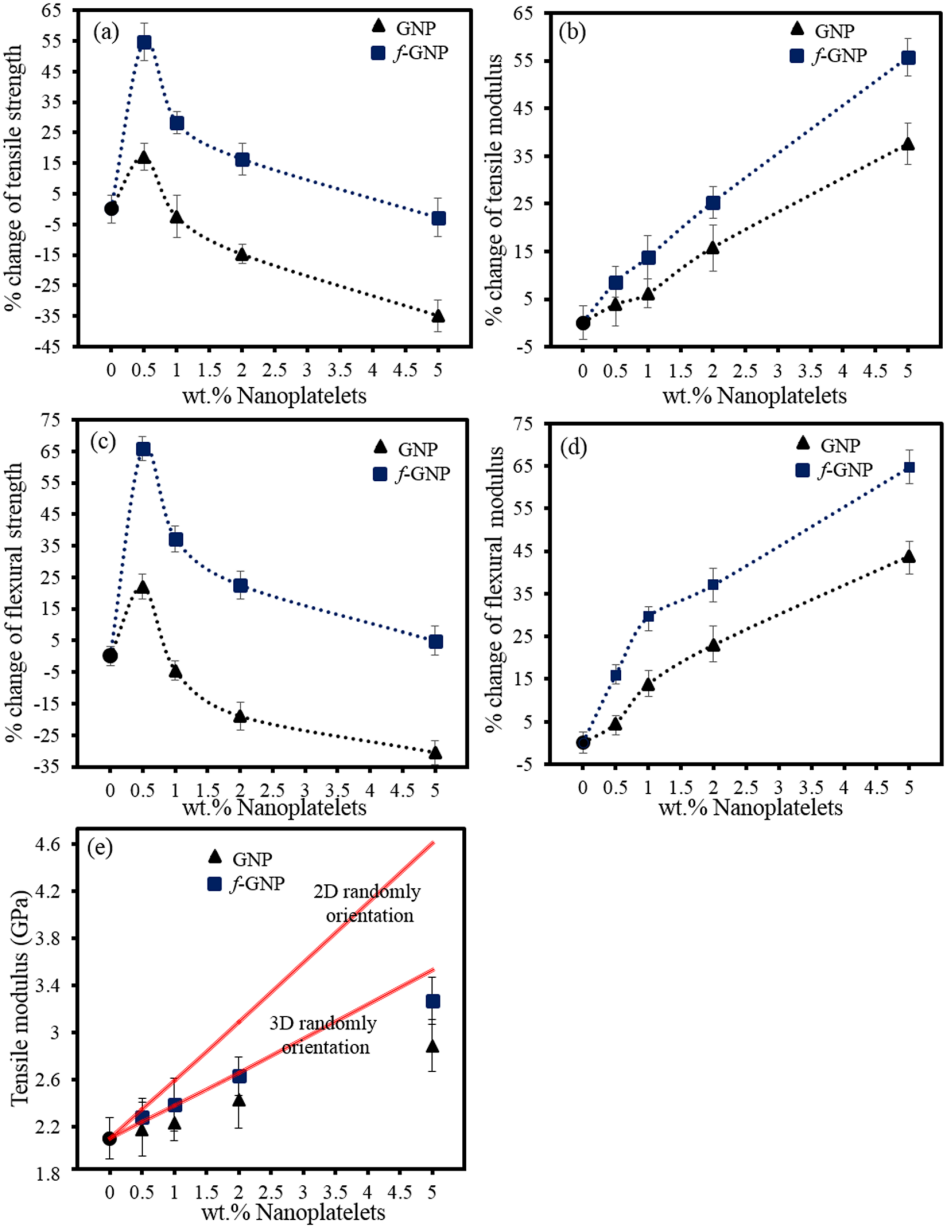
Changes percentage in various mechanical properties of nanocomposites compared to pure epoxy as a functional of nanoplatelets loadings (**a**–**d**), and comparison of the Halpin-Tsai model with experimental tensile modulus results (**e**).

The effects of GNP and *f*-GNP additions on mechanical behaviour of epoxy nanocomposites are summarized in Fig. 5a–d. It is noticed that nanocomposites strengths are more affected by change in interface adhesion, compared to nanocomposites moduli, which is due to the fact that the nanocomposites moduli are more controlled by the moduli and weight fractions of the nanocomposite constituents^53, 54^. The tensile and flexural moduli were increased with increasing nanoplatelets loadings, regardless of their physio-chemical functionalization. However, epoxy/*f*-GNP systems showed a more noticeable rise in moduli compared to the GNP at the same nanoplatelets content. The *f-*GNP reinforced nanocomposite with 5% loading showed about 55.7% and 64.6% increment in tensile and flexural moduli, respectively, over the pure epoxy, whereas the moduli of the GNP reinforced nanocomposite showed a lower increase with the same nanoplatelets content. The greater influence of functionalized graphite on improving the moduli of epoxy nanocomposites has been also reported by other researchers. It has been reported that, for example, amino-functionalization of GNP could increase the effective number of nanoplatelets incorporated into the matrix leading to a more homogeneous dispersion of nanoplatelets which in turn affects the nanocomposites moduli^55^.

We have also studied the distribution of nanoplatelets into epoxy matrix using Halpin-Tsai model. As shown in Fig. 5e, the 3D Halpin-Tsai model is well-fitted with experimental tensile moduli of epoxy/*f*-GNP nanocomposites compared to those related to GNP. In this regard, it is probably hypothesized that the physio-chemical functionalization of nanoplatelets, alongside the shearing effect of mechanical mixing, leads to formation of more bunches of oriented stacked nanoplatelets towards the through-plane direction. In other words, a more uniformly three-dimensional filler orientation was achieved which is associated to randomly arrangement of nanoplatelets. In comparison with the flexural properties, the addition of both GNP and *f*-GNP had less effect on tensile properties. This observation can be explained by 3D orientation of nanoplatelets into epoxy matrix. In other words, only 2D-aligned parallel planar distribution of nanoplatelets exhibited a more noticeable effect on tensile properties rather than 3D-random distribution^56^. According to the data presented in Table S1, compared with nanocomposites moduli, the tensile and flexural strengths of nanocomposites showed different trends. It is observed that the tensile and flexural strengths dramatically increase at a low GNP or *f*-GNP loading e.g. 0.5 wt%, and slightly decrease with increasing nanoplatelets loadings. At 0.5% loading of GNP, the nanocomposites showed 17% and 22% enhancement in the tensile strength and flexural strength, respectively. However, at the same loading of *f*-GNP, 54.6% and 65.8% increases in tensile strength and flexural strengths were observed due to possessing better stress transfer efficiency from matrix to nanoplatelets^57, 58^. Based on these results, compared to GNP, it can be hypothesised that the incorporation of *f*-GNP into epoxy matrix causes two positive synergistic effects on overall mechanical behaviour of nanocomposites. For instance, it can probably be deduced that the *f*-GNPs are capable of being mixed with epoxy resin uniformly since these materials possess some functional groups on their surface which can react with matrix and lead to formation of chemical bonds. Consequently, it can be postulated that *f*-GNPs have better affinity and compatibility towards epoxy resin in comparison with GNP.

Although the chemical bonding between matrix and *f*-GNP was formed via chemical functionalization on nanoplatelets, the separation and delamination of nanoplatelets, instead of the graphitic carbon–carbon bonding failure, could be taken place upon applied stress since nanoplatelets has weak interlayer forces (e.g. van der Waals forces). Consequently, the carbon–carbon bonding within the platelets remained intact. Additionally, as the content of GNP increases from 1% to 5% in the matrix, the GNP are prone to be more aggregated because of high surface area of nanoplatelets and strong π–π interactions resulted from the plane-to-plane contact of the neighbouring nanoplatelets^55, 59–61^. The last but not the least, imprisonment effect of nanoplatelets may deprive epoxy chains from being cross-linked, which is more obvious for higher loadings of GNP, (e.g. more than 1 wt%)^62–64^. In conclusion, these phenomena will lead to the formation of defects or flaws in the nanocomposites resulting in deterioration of strengths and poor exfoliation especially at higher nanoplatelets contents in comparison with lower nanoplatelets loadings. The improvements in mechanical properties and glass transition temperature obtained by inclusion of *f*-GNP in epoxy matrix is superior to what has been reported in the literatures for graphene reinforced epoxy composites. Table S2 comprehensively compares and summarizes the reported values published in literature for graphite nanocomposites.

#### Morphological properties

Regardless of its chemical functionalization, addition of graphite nanoplatelets into epoxy resin results in shifting of fracture mode form brittle to ductile at the low filler content, as illustrated in Fig. S2. In other words, except for a few large fracture steps, the fracture surface of pure epoxy (Fig. S2a) is almost smooth and flat exhibiting radiating ridge regions which is characteristics of a brittle fracture mode. However, the epoxy matrix reinforced with GNP (e.g. 0.5 wt%) possesses a large number of river structures leading to rougher and cleavage failure surface morphology (Fig. S2b), which can be attributed to nanoplatelets bridging effects. Nanoplatelets, especially *f*-GNP, can act as bridges and obstacles to delay crack initiation and propagation resulting in consumption of energy. Consequently, the crack path and direction are diverted or its tip may deflect which result in the fracture zones intercepted with each other with an angel around 90° (Fig. S2c), affecting mechanical properties and morphology^65^.

**Figure 6:**
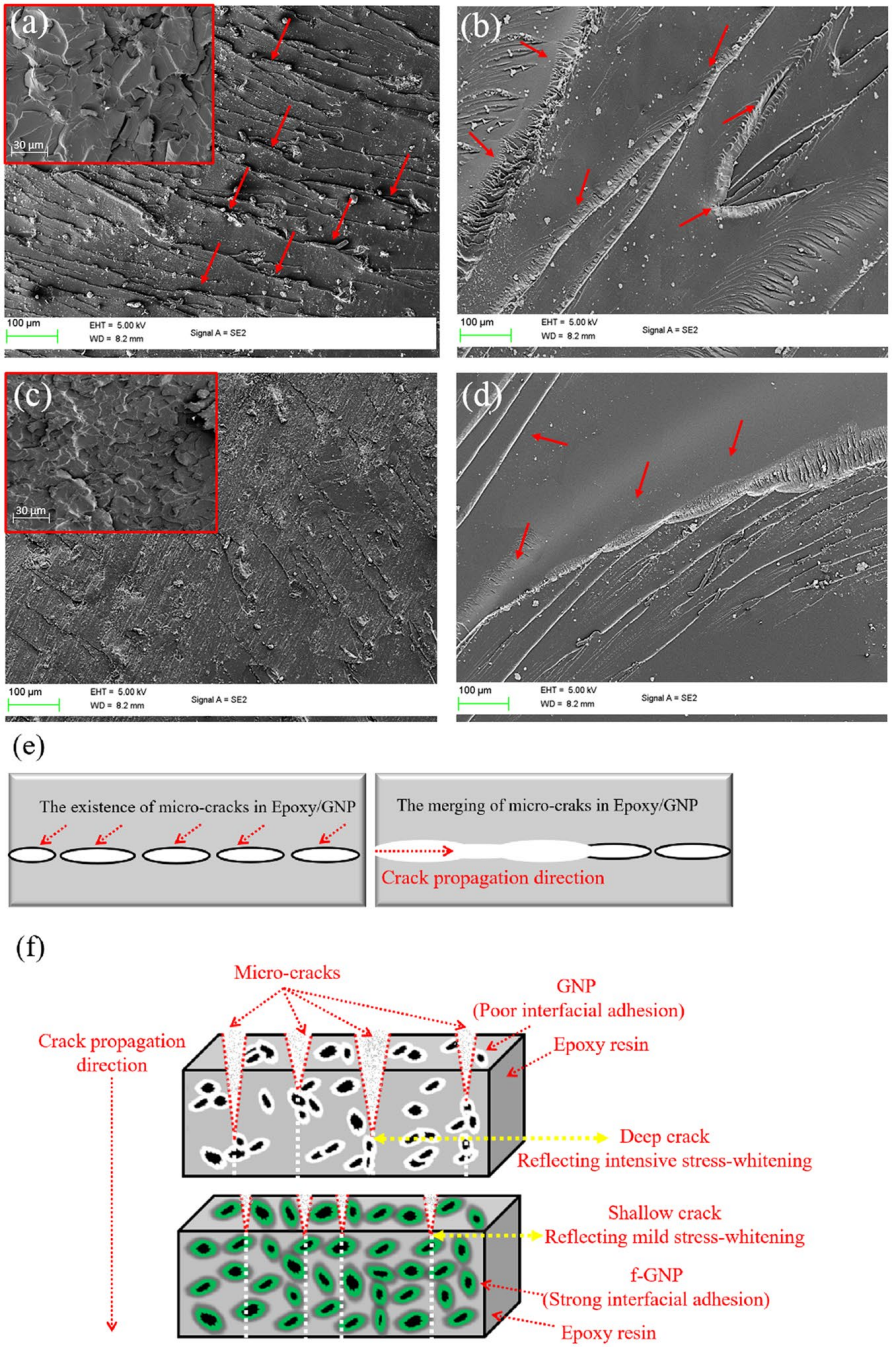
Micro-crack effect and stress-whitening phenomena (red arrows) in SEM images of epoxy/0.5% GNP nanocomposites (**a**,**b**) and epoxy/0.5% *f*-GNP nanocomposite at different magnifications (**c**,**d**) and a schematic of merging of micro-cracks in epoxy/GNP (**e**) and stress-whitening effect (**f**) in nanocomposites.

The procedure of crack deviations taken place during crack propagation has been considerably affected by the functionalization. Built-in micro-cracks in freeze-fractured surface of epoxy/GNP were more noticeable in numbers and size than epoxy/*f*-GNP nanocomposites according to Fig. 6a. In other words, as shown in Fig. 5c, the epoxy/*f*-GNP system has non-defective freeze-fractured surface attributed to higher surface energy, better compatibility and wettability of *f*-GNP to epoxy matrix than the unmodified GNP. Compared with epoxy/*f*-GNP, when the stress was applied to the epoxy/GNP nanocomposites, these inherent mentioned micro-cracks could merge together to form a larger crack resulting in easier crack propagation (Fig. 6e). Consequently, as illustrated in magnified sections of Fig. 6a and c, and despite the rough fracture surface of both epoxy/*f*-GNP and epoxy/GNP nanocomposites, the epoxy/GNP nanocomposites possesses larger fracture districts than epoxy/*f*-GNP nanocomposite. In other words, catastrophic failure ascribe to heterogeneity in system can be seen in epoxy/GNP samples and the crack deviation is less tangible in such materials Furthermore, voids at filler-matrix interface as well as agglomeration can highlight such phenomenon in samples. This can be also explained by stress-whitening effect. As it is presented in Fig. 6b and d, the stress-whitening phenomena, as an evident of existence of micro-crack, is more profound for epoxy/GNP system. According to Fig. 6f, deep penetrated cracks can be seen for samples consisting GNP, whereas shallow ones can be observed for samples comprising *f*-GNP. It can be postulated that such variation in depth of cracks arising from micro-crack and poor interfacial adhesion can probably highlight the stress-whitening effect. Therefore, the addition of nanoplatelets, especially unmodified type and at high level of loadings, could have adverse effects on mechanical properties of the composites.

**Figure 7:**
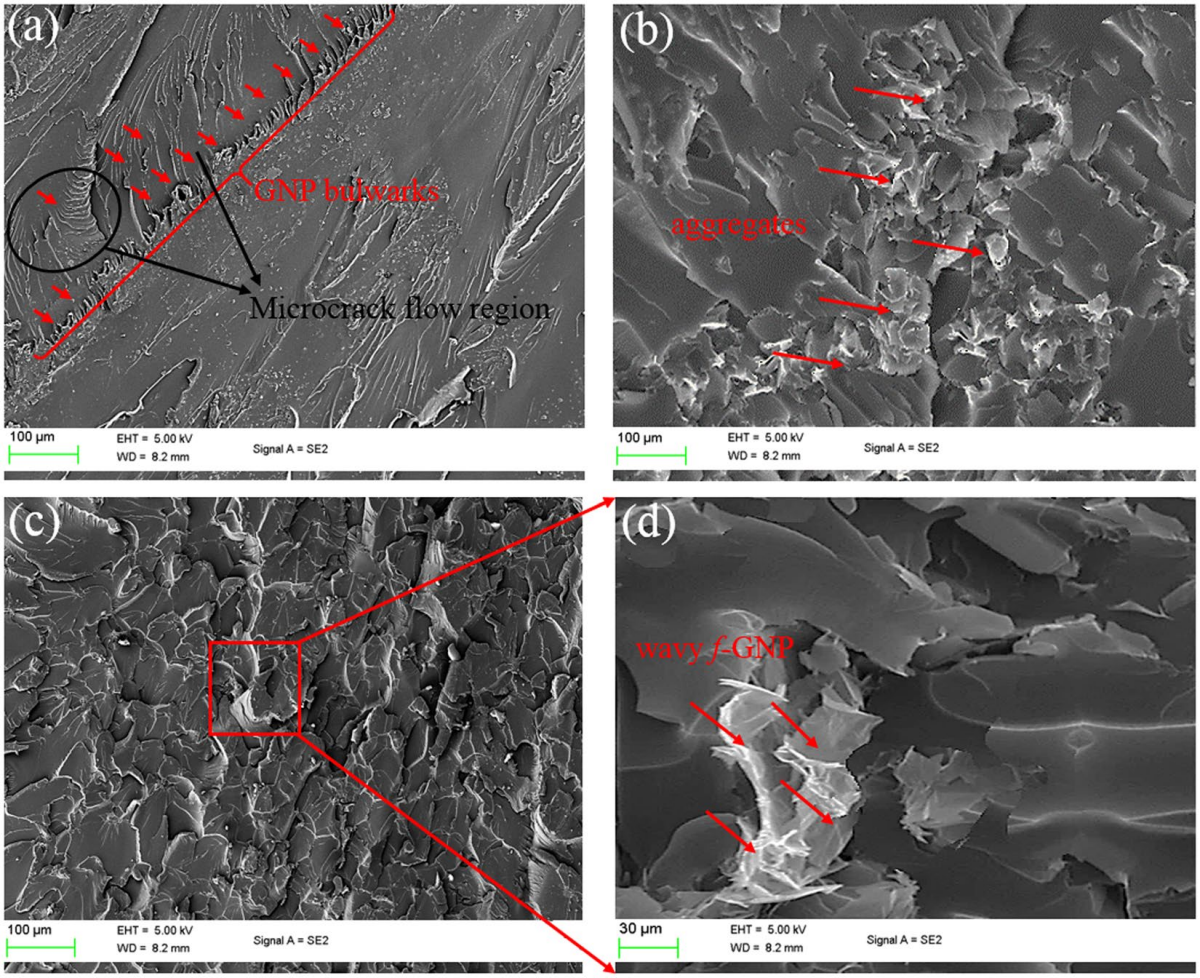
SEM images of fracture surfaces of epoxy/0.5% GNP (**a**,**b**) and epoxy/0.5% *f*-GNP (**c**,**d**) nanocomposites at different magnifications.

Moreover, as shown in Fig. 7a, the addition of low content of the nanoplatelets into epoxy can act like propagation-retarding bulwarks being perpendicular to micro-crack flow. Consequently, the crack energy will be consumed and dissipated and either break through or pull out nanoplatelets from matrix, whereas due to the poor interfacial adhesion between GNP and epoxy, nanoplatelets could easily be pulled out from the matrix and the crack propagates on the epoxy-nanoplatelets interface. In contrast, chemical covalent bondings between *f*-GNP and matrix would highlight the bridging effect of nanoplatelets leading to crack propagation in the bulk matrix instead of interfacial region which leads to the improvement of mechanical properties^55, 66–68^. In other words, the applied stress is set to be consumed to overcome the strong interfacial adhesion between *f*-GNP and matrix and the *f*-GNP as long as physical and chemical anchors were formed at interface. Following that, deflected and torturous crack propagation arising from such hindrances, these strong attached bridges, were observed in samples.

In comparison with epoxy/*f*-GNP nanocomposites (Fig. 7c), large agglomerations of GNP exist in epoxy matrix (Fig. 7b). Whereas the considerable cleavage surface of epoxy/*f*-GNP (Fig. 7c) shows uniform distribution and dispersion of nanoplatelets within the matrix so that it could significantly change the crack directions. It is proposed that the uniform dispersion is arisen from (a) creation of functional groups which can form strong chemical covalent bonds with epoxy matrix and improve wettability of nanoplatelets to resin; and (b) prevention of re-agglomeration of nanoplatelets^57, 69, 70^. Moreover, it is postulated that after amino-functionalization of GNP, the surface of those pulled out *f*-GNP cannot remain intact since the chemical bonding between *f*-GNPs and epoxy formed (Fig. 7d), which can also lead to mechanical locking effect, improving resistance against the pull-out forces. In spite of the amino-functionalization and high-energy sonication, some inevitable agglomerates still existat the higher *f*-GNP content (e.g. 5%.), indicating that the nanoplatelets could not be dispersed well, and the agglomerated nanoplatelets and their cluster can act as stress concentration sites after entering the plastic regimes leading to earlier catastrophic fracture and reduction of nanocomposite strengths (see Fig. S3)^55, 71, 72^.

## Conclusion

In the first part, “*graphite collision-induced activation”* term refers exposing the graphite nanoplatelets to the high collision force generated by a simple kitchen blender, which results in creating electron-active sites. These electron-active sites have been used for grafting the maleic anhydride as a dienophile specie, which mechanistically follows Diels-Alder chemistry. The behaviour of the collision-induced activation of graphite investigated by DFT studies showed that uniformly distributed π-electrons of graphite produce an induced electron flow towards the edges. Chemical interaction of the collision-induced activated graphite with maleic anhydride was approved by FTIR, Raman, ^13^C-NMR, and XPS spectroscopy, indicating a 12 fold increase in O/C ratio in comparison to pure graphite nanoplatelets calculated by XPS analysis. The key points of this new method are being the solvent-free and scalable process which enables industrial adoption of this method. Although we utilized the grafted maleic anhydrides on graphite nanoplatelets for further amino-functionalization for reinforcement of polymer nanocomposites used in aerospace applications, these generated sites can be easily used as electrophilic sites to introduce various functional groups for other applications e.g. sensor, biomedical, and etc. In the second part, the amino-functionalized graphite nanoplatelets at various percentages were loaded into epoxy polymer to study its influence on the thermo-physical properties of composites in relation with their interfacial interactions. The *f*-GNPs exhibited a uniform dispersion being capable of producing a 3D structure in matrix which was also confirmed by Halpin-Tsia model. Furthermore, the effective stress transfer in nanocomposites containing functionalised graphite nanoplatelets indicates the possibility of incorporations of higher concentration of nanoplatelets without running into formation of agglomerates. According to the morphological studies, as crack is initiated or propagated, it might encounter the nanoplatelets. Crack energy is then consumed passing through nanoplatelets. In such condition, the graphite nanoplatelets could easily be passed by and pulled out from the matrix. In contrast, chemically bonded *f*-GNPs to the polymer matrix will not be easily pulled out. Consequently, much more energy dissipated resulted in better mechanical properties.

## Materials and Methods

For detailed materials, general experiments, and measurements, see the supplementary information.

### Computational details

Model: graphene model is made of carbon atoms arranged on a honeycomb structure with hexagons and can be considered as composed of benzene rings stripped out from their hydrogen atoms^73^. We worked on an array of graphite nanopatlates containing 45 carbon atoms with 14 benzene rings, in which the carbon atoms were terminated with the hydrogen atoms.

Method***:*** Geometry of all structures were optimized through B97-D/6 − 31 + G* level of DFT-D approach implemented in Gaussian 09 package^74^. The frequency calculations were performed for all optimized structures in order to ensure being in real minimum. To emphasize the importance of dispersion energy as a key factor in stabilizing the stacked dimers, dispersion-corrected DFT functional (B97-D) was selected. Recently, B97-D has been widely used to describe macro structures like graphene sheets^75–78^. In the study of intermolecular interactions counterpoise (CP) correction^79, 80^ was performed to limit the basis set superposition error (BSSE). The binding energy (*E*_*bind*_), which is indicative of the thermodynamic stability of the system, was evaluated by the subtraction of the total energy of the complex, *E*_*complex*_, from sum of its constituent parts, *E*_*fragment*_:

2 $${E}_{bind}={E}_{complex}-({\sum }^{}{E}_{fragment})$$

All structures were in their lowest energy state.

### Blender-assisted functionalization of GNP with MA (m-GNP)

In a typical solvent-free process, a powder mixture of pre-washed GNP (5 g) and maleic anhydride (3 g) was loaded into a kitchen blender (1000 W, 25 Hz) equipped with a six-pointed star shaped blade (see Fig. 1) and then was placed into a glove-box pre-filled with a continuous nitrogen flow. Afterwards, the blender was run for 12 cycles of 10 min at ~5000 rpm at room temperature. The resulting powder was then dispersed in acetone (200 ml) and stirred for 30 min to dissolve un-reacted maleic anhydride. The total mixture was filtered and washed several times with acetone and then was stored under vacuum at 50 °C for further use. As a control sample (*c*-GNP), pure GNP without maleic anhydride was processed following the same above-mentioned procedure.

### Amino-functionalization of m-GNP (f-GNP)

For amino-functionalization, *m*-GNP (1 g) was dispersed into 250 ml solution of tetraethylenepentamine (TEPA) in dimethylformamide (DMF) (25:75 w/w) and the total mixture was sonicated for 30 min before stirring at 100 °C for 18 h under reflux condition. After cooling to room temperature, the resulting solution was firstly filtered under vacuum and then rinsed several times with acetone to remove the un-reacted chemicals.

### Epoxy nanocomposites preparation

To fabricate the nanocomposites, specific percentages of various graphite nanoplatelets were added into the epoxy resin and the suspensions were stirred for 3 h using a magnetic stirrer plate at 70 °C. As reported, if very small amount of solvent as diluents e.g. acetone remains in epoxy polymer suspension, it strongly affects properties of epoxy matrix. Thus, instead of use of the solvent for dispersion of nanoplatelets into epoxy resin which is also industrially inaccessible, a Hielscher UIP1000-230 ultrasonic processor operating at a frequency of 15 kHz was used to generate high-energy ultrasonic waves with an amplitude of 80 μm peak-to-peak through the epoxy suspensions for 60 min with an ultrasonic pulsing cycle of 2 s on and 2 s off. Then, a stoichiometric ratio of hardener was added to the mixtures before degasify the bubbles under vacuum for 30 min. The final mixtures then was poured into a mould and curing was completed in an oven at 70 °C for 6 h, and then at 100 °C for 1.5 h.

## Electronic supplementary material

SUPPLEMENTARY INFO

## References

[1] Zhao W (2010). Preparation of graphene by exfoliation of graphite using wet ball milling. Journal of Materials Chemistry.

[2] Zhu Y (2010). Graphene and Graphene Oxide: Synthesis, Properties, and Applications. Advanced Materials.

[3] Edwards RS, Coleman KS (2013). Graphene synthesis: relationship to applications. Nanoscale.

[4] Arao Y, Mizuno Y, Araki K, Kubouchi M (2016). Mass production of high-aspect-ratio few-layer-graphene by high-speed laminar flow. Carbon.

[5] Du W, Jiang X, Zhu L (2013). From graphite to graphene: direct liquid-phase exfoliation of graphite to produce single- and few-layered pristine graphene. Journal of Materials Chemistry A.

[6] Ciesielski A, Samori P (2014). Graphene via sonication assisted liquid-phase exfoliation. Chemical Society Reviews.

[7] Guardia L (2011). High-throughput production of pristine graphene in an aqueous dispersion assisted by non-ionic surfactants. Carbon.

[8] Yi M, Shen Z (2015). A review on mechanical exfoliation for the scalable production of graphene. Journal of Materials Chemistry A.

[9] Arao Y, Kubouchi M (2015). High-rate production of few-layer graphene by high-power probe sonication. Carbon.

[10] Yi M, Shen Z (2014). Kitchen blender for producing high-quality few-layer graphene. Carbon.

[11] Varrla E (2014). Turbulence-assisted shear exfoliation of graphene using household detergent and a kitchen blender. Nanoscale.

[12] Tour JM (2014). Layered materials: Scaling up exfoliation. Nat Mater.

[13] Zaman I (2011). Epoxy/graphene platelets nanocomposites with two levels of interface strength. Polymer.

[14] Wolf, E. L. In *Applications of Graphene: An Overview* 19–38 (Springer International Publishing, 2014).

[15] Park YT (2015). Epoxy Toughening with Low Graphene Loading. Advanced Functional Materials.

[16] Zaman I (2012). A Facile Approach to Chemically Modified Graphene and its Polymer Nanocomposites. Advanced Functional Materials.

[17] Chen Y (2016). High-Performance Epoxy Nanocomposites Reinforced with Three-Dimensional Carbon Nanotube Sponge for Electromagnetic Interference Shielding. Advanced Functional Materials.

[18] Zabihi O, Omrani A, Rostami AA (2012). Thermo-oxidative degradation kinetics and mechanism of the system epoxy nanocomposite reinforced with nano-Al2O3. Journal of Thermal Analysis and Calorimetry.

[19] Zabihi O (2016). One-step amino-functionalization of milled carbon fibre for enhancement of thermo-physical properties of epoxy composites. Composites Part A: Applied Science and Manufacturing.

[20] Li Z (2013). Control of the functionality of graphene oxide for its application in epoxy nanocomposites. Polymer.

[21] Wei J, Vo T, Inam F (2015). Epoxy/graphene nanocomposites - processing and properties: a review. RSC Advances.

[22] Potts JR, Dreyer DR, Bielawski CW, Ruoff RS (2011). Graphene-based polymer nanocomposites. Polymer.

[23] Yue L, Pircheraghi G, Monemian SA, Manas-Zloczower I (2014). Epoxy composites with carbon nanotubes and graphene nanoplatelets – Dispersion and synergy effects. Carbon.

[24] Lv C (2010). Effect of Chemisorption on the Interfacial Bonding Characteristics of Graphene−Polymer Composites. The Journal of Physical Chemistry C.

[25] Kim K-S, Jeon I-Y, Ahn S-N, Kwon Y-D, Baek J-B (2011). Edge-functionalized graphene-like platelets as a co-curing agent and a nanoscale additive to epoxy resin. Journal of Materials Chemistry.

[26] Kuila T (2012). Chemical functionalization of graphene and its applications. Progress in Materials Science.

[27] Liu J, Tang J, Gooding JJ (2012). Strategies for chemical modification of graphene and applications of chemically modified graphene. Journal of Materials Chemistry.

[28] Dreyer DR, Todd AD, Bielawski CW (2014). Harnessing the chemistry of graphene oxide. Chemical Society Reviews.

[29] Horbatenko Y, Choi M, Ruoff RS, Bielawski CW, Park N (2015). First-principles investigation of wet-chemical routes for the hydrogenation of graphene. Carbon.

[30] He F, Lam K-H, Fan J, Chan LH (2014). Improved dielectric properties for chemically functionalized exfoliated graphite nanoplates/syndiotactic polystyrene composites prepared by a solution-blending method. Carbon.

[31] Zhong X (2010). Aryne cycloaddition: highly efficient chemical modification of graphene. Chemical Communications.

[32] He H, Gao C (2010). General Approach to Individually Dispersed, Highly Soluble, and Conductive Graphene Nanosheets Functionalized by Nitrene Chemistry. Chemistry of Materials.

[33] Naebe M (2014). Mechanical Property and Structure of Covalent Functionalised Graphene/Epoxy Nanocomposites. Scientific Reports.

[34] Kaper H, Grandjean A, Weidenthaler C, Schüth F, Goettmann F (2012). Surface Diels–Alder Reactions as an Effective Method to Synthesize Functional Carbon Materials. Chemistry – A European Journal.

[35] Sarkar S, Bekyarova E, Niyogi S, Haddon RC (2011). Diels−Alder Chemistry of Graphite and Graphene: Graphene as Diene and Dienophile. Journal of the American Chemical Society.

[36] Sarkar S, Bekyarova E, Haddon RC (2012). Chemistry at the Dirac Point: Diels–Alder Reactivity of Graphene. Accounts of Chemical Research.

[37] Seo J-M, Jeon I-Y, Baek J-B (2013). Mechanochemically driven solid-state Diels-Alder reaction of graphite into graphene nanoplatelets. Chemical Science.

[38] Ji Z, Chen J, Huang L, Shi G (2015). High-yield production of highly conductive graphene via reversible covalent chemistry. Chemical Communications.

[39] Frolova LV (2015). Tetracyanoethylene oxide-functionalized graphene and graphite characterized by Raman and Auger spectroscopy. Carbon.

[40] Bandurin DA (2016). Negative local resistance caused by viscous electron backflow in graphene. Science.

[41] Ivanciuc O, Klein DJ, Bytautas L (2002). Unpaired π-spin density in defected graphite. Carbon.

[42] Baturin S, Kanareykin A (2014). Cherenkov Radiation from Short Relativistic Bunches: General Approach. Physical review letters.

[43] Akhmediev N, Karlsson M (1995). Cherenkov radiation emitted by solitons in optical fibers. Physical Review A.

[44] Grant, I. S. & Phillips, W. R. *Electromagnetism* (John Wiley & Sons, 2013).

[45] Dennington, R. D., Keith, T. A. & Millam, J. M. GaussView 5.0. 8. *Gaussian Inc* (2008).

[46] Jeon I-Y, Bae S-Y, Seo J-M, Baek J-B (2015). Scalable Production of Edge-Functionalized Graphene Nanoplatelets via Mechanochemical Ball-Milling. Advanced Functional Materials.

[47] Jeon I-Y (2013). Large-Scale Production of Edge-Selectively Functionalized Graphene Nanoplatelets via Ball Milling and Their Use as Metal-Free Electrocatalysts for Oxygen Reduction Reaction. Journal of the American Chemical Society.

[48] Wang F, Drzal LT, Qin Y, Huang Z (2016). Enhancement of fracture toughness, mechanical and thermal properties of rubber/epoxy composites by incorporation of graphene nanoplatelets. Composites Part A: Applied Science and Manufacturing.

[49] Kudin KN (2008). Raman Spectra of Graphite Oxide and Functionalized Graphene Sheets. Nano Letters.

[50] Cançado LG, Pimenta MA, Neves BRA, Dantas MSS, Jorio A (2004). Influence of the Atomic Structure on the Raman Spectra of Graphite Edges. Physical Review Letters.

[51] Ferrari AC (2007). Raman spectroscopy of graphene and graphite: Disorder, electron–phonon coupling, doping and nonadiabatic effects. Solid State Communications.

[52] Seo J-M, Jeon I-Y, Baek J-B (2013). Mechanochemically driven solid-state Diels-Alder reaction of graphite into graphene nanoplatelets. Chemical Science.

[53] Li J, Kim J-K, Lung Sham M (2005). Conductive graphite nanoplatelet/epoxy nanocomposites: Effects of exfoliation and UV/ozone treatment of graphite. Scripta Materialia.

[54] Zabihi O, Khayyam H, Fox BL, Naebe M (2015). Enhanced thermal stability and lifetime of epoxy nanocomposites using covalently functionalized clay: experimental and modelling. New Journal of Chemistry.

[55] Wang F, Drzal L, Qin Y, Huang Z (2015). Mechanical properties and thermal conductivity of graphene nanoplatelet/epoxy composites. Journal of Materials Science.

[56] Huang T (2012). Chemically Modified Graphene/Polyimide Composite Films Based on Utilization of Covalent Bonding and Oriented Distribution. ACS Applied Materials & Interfaces.

[57] Ma J (2013). Covalently bonded interfaces for polymer/graphene composites. Journal of Materials Chemistry A.

[58] Zabihi O (2013). Preparation and characterization of toughened composites of epoxy/poly(3,4-ethylenedioxythiophene) nanotube: Thermal, mechanical and electrical properties. Composites Part B: Engineering.

[59] Li W, Dichiara A, Bai J (2013). Carbon nanotube–graphene nanoplatelet hybrids as high-performance multifunctional reinforcements in epoxy composites. Composites Science and Technology.

[60] Zabihi O, Ahmadi M, Khayyam H, Naebe M (2016). Fish DNA-modified clays: Towards highly flame retardant polymer nanocomposite with improved interfacial and mechanical performance. Scientific Reports.

[61] Zabihi O, Ahmadi M, Naebe M (2017). Self-assembly of quaternized chitosan nanoparticles within nanoclay layers for enhancement of interfacial properties in toughened polymer nanocomposites. Materials & Design.

[62] Zhang Y (2014). Tuning the interface of graphene platelets/epoxy composites by the covalent grafting of polybenzimidazole. Polymer.

[63] Liu W (2012). Simultaneous catalyzing and reinforcing effects of imidazole-functionalized graphene in anhydride-cured epoxies. Journal of Materials Chemistry.

[64] Zabihi O, Khodabandeh A, Ghasemlou S (2012). Investigation of mechanical properties and cure behavior of DGEBA/nano-Fe2O3 with polyamine dendrimer. Polymer Degradation and Stability.

[65] Zaman I, Manshoor B, Khalid A, Araby S (2014). From clay to graphene for polymer nanocomposites—a survey. J Polym Res.

[66] Gojny FH, Wichmann MHG, Fiedler B, Schulte K (2005). Influence of different carbon nanotubes on the mechanical properties of epoxy matrix composites – A comparative study. Composites Science and Technology.

[67] Shokrieh MM, Esmkhani M, Shahverdi HR, Vahedi F (2013). Effect of Graphene Nanosheets (GNS) and Graphite Nanoplatelets (GNP) on the Mechanical Properties of Epoxy Nanocomposites. Science of Advanced Materials.

[68] Zabihi O (2012). Modeling of phenomenological mechanisms during thermal formation and degradation of an epoxy-based nanocomposite. Thermochimica Acta.

[69] Zaldivar R, Adams P, Kim H, Nokes J (2015). Mechanical enhancement of graphite nanoplatelet composites: Effect of matrix material on the atmospheric plasma-treated GnP reinforcement. Journal of Composite Materials.

[70] Zabihi O, Ghasemlou S (2012). Nano-CuO/Epoxy Composites: Thermal Characterization and Thermo-Oxidative Degradation. International Journal of Polymer Analysis and Characterization.

[71] Li B, Zhong W-H (2011). Review on polymer/graphite nanoplatelet nanocomposites. Journal of Materials Science.

[72] Yasmin A, Daniel IM (2004). Mechanical and thermal properties of graphite platelet/epoxy composites. Polymer.

[73] Zhang S (2010). First-Principles Study of Field Emission Properties of Graphene-ZnO Nanocomposite. The Journal of Physical Chemistry C.

[74] Frisch, M. *et al*. Gaussian 09, Revision A. 02, Gaussian. *Inc*., *Wallingford*, *CT* **200** (2009).

[75] Grimme S (2011). Density functional theory with London dispersion corrections. Wiley Interdisciplinary Reviews: Computational Molecular Science.

[76] Becke AD (1993). Density‐functional thermochemistry. III. The role of exact exchange. The Journal of chemical physics.

[77] Antony J, Grimme S (2006). Density functional theory including dispersion corrections for intermolecular interactions in a large benchmark set of biologically relevant molecules. Physical Chemistry Chemical Physics.

[78] Peverati R, Baldridge KK (2010). Implementation and Performance of DFT-D with Respect to Basis Set and Functional for Study of Dispersion Interactions in Nanoscale Aromatic Hydrocarbons. Journal of chemical theory and computation.

[79] Schwenke DW, Truhlar DG (1985). Systematic study of basis set superposition errors in the calculated interaction energy of two HF molecules. The Journal of chemical physics.

[80] Boys S, Bernardi F (2002). The calculation of small molecular interactions by the differences of separate total energies. Some procedures with reduced errors. Molecular Physics.

