# Supplementary material for: Collision-induced activation: Towards industrially scalable approach to graphite nanoplatelets functionalization for superior polymer nanocomposites
Source: Sci Rep. 2017 Jun 15;7:3560. doi: 10.1038/s41598-017-03890-8 (PMC5472569; doi:10.1038/s41598-017-03890-8)
Supplement: Supplementary file 1 — SUPPLEMENTARY INFO [file 41598_2017_3890_MOESM1_ESM.pdf]

Supplementary Information

Collision-induced activation: Towards industrially scalable approach to graphite nanoplatelets functionalization for superior polymer nanocomposites

Omid Zabihi*[,1], Mojtaba Ahmadi[2], Tahereh Abdollahi[3], Saeid Nikafshar[4], Minoo Naebe*[,1]

[1]Deakin University, Geelong, Australia, Carbon Nexus, Institute for Frontier Materials
[2]Department of Chemical Engineering, Isfahan University of Technology, Isfahan, 84156/83111, Iran
[3]Department of Physical Chemistry, University of Mazandaran, Babolsar, Iran
[4]Department of Applied Chemistry, Faculty of Chemistry, University of Tabriz, Tabriz, Iran

Contents:

1. Materials and general experimentations	Page S2
2. Measurements	Page S2
3. Grafting estimation	Page S5
Fig. S1. TGA thermograms of GNP, *m*-GNP and *f*-GNP at heating rate of 10 ^{o}C/min under nitrogen atmosphere.	Page S5
Table S1. Mechanical properties of epoxy nanocomposites containing various contents of GNP and *f*-GNP.	Page S6
Table S2. Summary of the effect of different graphene chemical treatments on the tensile, the flexural properties, and glass transition temperature in several nanocomposites containing low loadings of graphene (e.g. 0.5 %wt) as reported in the literature.	Page S7
Fig. S2. SEM images of fracture surfaces of pure epoxy (a) and epoxy/0.5% GNP nanocomposites (b & c).	Page S12
Fig. S3. SEM image of fracture surface of epoxy/5% *f*-GNP nanocomposites containing high level of agglomerations (red arrow).	Page S12
References	Page S13

*Corresponding author: Tel: +61469570372
E-mail: Omid.Zabihi@deakin.edu.au
*Corresponding author: Tel: +61352271410
E-mail: Minoo.Naebe@deakin.edu.au

1. Materials and general experimentations

Graphite nanoplatelets (grade C, with a surface area of ~518 m^2/g as measured by BET) was supplied as bulk dry powder by XG Sciences' xGnP®, Michigan, USA, which typically consist of aggregates of sub-micron platelets having a particle diameter of <10 μm and a thickness of less than a few nanometers. The received graphite nanoplatelets were refluxed in deionized water/ethanol solution for 72 h and then filtered and well-rinsed. The washed graphite nanoplatelets was then heated for 3 min at 700 ^oC under argon atmosphere before storing in a vacuum oven at 100 ^oC to remove any impurities and moisture prior to use. Maleic anhydride (98%) was obtained from Sigma and used as received. An epoxy resin of DER 332 having equivalent weight of 175 g/eq under trademark of The Dow Chemical Company and hardener of tetraethylenepentamine was used as thermosetting epoxy polymer system (Sigma-Aldrich). All solvents used in this study were of analytical grade.

2. Measurements

Brunauer–Emmett–Teller (BET) surface area was measured by a Micromeritics TriStar 3000 using adsorption isotherm of nitrogen at 77 K and Malvern Mastersizer 2000 (UK) was used for determining particle sizes. Deionized water was used as a dispersion media for particle size measurements. The reflective index of 2.42 was used in the calculation of particle size distribution of graphene nanopatlates. Each experiment was carried out three times and error bars were not determined as the differences were insignificant. Fourier transform infrared (FTIR) analysis was performed by a Bruker Vertex 70 FTIR spectrometer in ATR mode with a resolution of 4 cm^{-1}. Raman measurements were conducted using a Renishaw InVia Raman Microspectrometer (Renishaw, Gloucestershire, UK) with diode laser at 514 nm at room temperature. All crbon-13 nuclear magnetic resonance (^{13}C-NMR) measurements were done on a Bruker AVANCE III NMR spectrometer operating at 75.4 MHz. Samples were packed into 4mm zirconia MAS rotors and magic-angle-spinning (MAS) at 10 kHz was used. ^{13}C measurements with direct carbon excitation and proton decoupling during acquisition were done to get comparable carbon spectra. For these a ^{13}C excitation 90° pulse of 3 microseconds was used, applying a recycle delay of 24 sec for 6k sampled scans, totaling in 51 h measurement time per sample. X-ray photoelectron spectroscopy (XPS) analysis was performed using an AXIS Nova spectrometer (Kratos Analytical Inc., Manchester, UK) with a monochromated Al K_α source at a power of 180 W (15 kV × 12 mA) and a hemispherical analyser operating in the fixed analyser transmission mode. The total pressure in the main vacuum chamber during analysis was typically 10^{-8} mbar. Survey spectra were acquired at a pass energy of 160 eV. To obtain more detailed

information about chemical structure, oxidation states etc., high resolution spectra were recorded from individual peaks at 20 eV pass energy (yielding a typical peak width for polymers of 0.8 - 1.0 eV). All elements present were identified from survey spectra. The atomic concentrations of the detected elements were calculated using integral peak intensities and the sensitivity factors supplied by the manufacturer. Binding energies were referenced to the C 1s peak at 284.5 eV for graphitic carbon. Precision (i.e. reproducibility) depends on the signal/noise ratio but is usually much better than 5%. The latter is relevant when comparing similar samples. Water contact angles were measured using a KSV Model CAM101 Contact Angle Meter (KSV Instruments Ltd, Finland) equipped with an Olympus DP70 high resolution microscope. The image of each drop was captured after 0.1 seconds after deposition onto sample surface in ambient conditions. Thermogravimetry analysis (TGA) experiments were carried out using a TA Instruments Q50 TGA, which were conducted in nitrogen atmosphere at a heating rate of 10 ^{o}C/min from room temperature to 750 ^{o}C. Dynamic mechanical properties of the produced materials were measured using a TA Instruments Q800 DMA. The samples were polished to ~30mm×10mm×1 mm before being mounted on a single cantilever clamp. The DMTA tests experimental conditions were: temperature range, 25–250 ^{o}C; frequency of 1 Hz; and heating rate 5 ^{o}C/min. The tensile properties were measured by using an Instron Universal machine at cross-head speed of 5 mm/min according to ASTM D638. The tensile strengths and Young's moduli of the nanocomposites were measured. In addition, the Halpin–Tsai model as a micromechanical model was employed to predict the tensile modulus of epoxy nanocomposites. This model links the modulus of a nanocomposite to constituent properties, concentrations of each constituent, as well as aspect ratio and orientation of the filler. According to this model, the composite tensile modulus (E_C) can be predicted by the following equations [1-4].

$$E_c = \left[0.375\left(\frac{1+\xi\eta_L V_f}{1-\eta_L V_f} \right) + 0.625\left(\frac{1+2\eta_T V_f}{1-\eta_T V_f} \right) \right] E_m$$

2D Randomly oriented filler (1)

$$E_c = \left[0.2\left(\frac{1+\xi\eta_L V_f}{1-\eta_L V_f} \right) + 0.8\left(\frac{1+2\eta_T V_f}{1-\eta_T V_f} \right) \right] E_m$$

3D Randomly oriented filler (2)

Where the η_T and η_L parameters can be calculated by following equations:

$$\eta_T = \frac{\left(\frac{E_f}{E_m}\right) - 1}{\left(\frac{E_f}{E_m}\right) + 2}\ ;\ \eta_L = \frac{\left(\frac{E_f}{E_m}\right) - 1}{\left(\frac{E_f}{E_m}\right) + \xi} \tag{3}$$

For all the above formulations, ξ, $V_{f,}$, E_m, and E_f are shape factor, volume fraction of filler, tensile modulus of matrix, and tensile modulus of filler, respectively. For the Halpin-Tsai model, the tensile modulus of GNPs, E_f, was equal to the modulus of exfoliation in the graphite c-axis (through-the-plane) in the order of 36.5 GPa and the filler shape factor, ξ, is equal to 0.667 (L/d) for platelets [3-6].

In order to study flexural strength and modulus, 3-point bending test also were conducted according to ASTM D790-02 using an Instron universal testing machine. The tests were performed with a 10 kN load cell at a cross-head speed of 2 mm/min and the span-to-depth ratio was maintained at 16:1. The maximum flexural stress at failure on the tension side of a flexural sample, calculated from Eq. 4, was considered as the flexural strength (S) of the material. Moreover, flexural modulus (E) was determined from the slope (m) of the initial straight-line portion of the load-deflection curve according to Eq. 5.

$$S = 3PL / 2bd^2 \tag{4}$$

$$E = L^3 m / 4bd^3 \tag{5}$$

Where P is the applied load at the deflection point, b, d and L are the width, depth of the samples and the span length, correspondingly. At least five specimens from each sample were tested and the average data and standard deviation of strengths and moduli were reported.

The morphology of nanocomposites was studied by scanning electron microscopy of mechanical-fractured and freeze-fractured surfaces. For the mechanical-fractured surfaces, the samples were fractured during mechanical test and the freeze-fractured surfaces were manually prepared by cooling in liquid nitrogen. The obtained fracture surfaces were sputter-coated with gold. The SEM observations were obtained with a Philips field emission gun SEM (model: XL30, Netherlands) operated at 25 kV.

3. Grafting estimation

Surface functionalization degree could be approximately quantified using the following equation [7,8]:

$$\text{attached amount (mmol/g)} = \frac{1000\Delta W}{(100-\Delta W)M} \quad (6)$$

Where ΔW is difference of %weight loss for pure GNP with *m*-GNP during degradation process at an inert atmosphere, up to 600 ^{o}C, which is the temperature, assuming that graphitic structure of graphite nanoplatelets remains untouched and existing weight loss only results from the molecules on the surface. The M is the molecular weight of maleic anhydride. For our graphene nanoplatelets systems, ΔW and M are ~8.5% and 98.06 g/mol, respectively. Accordingly, ~0.94 mmol maleic anhydride equaled to 92.1 mg was obtained to be attached on per gram of pure GNP.

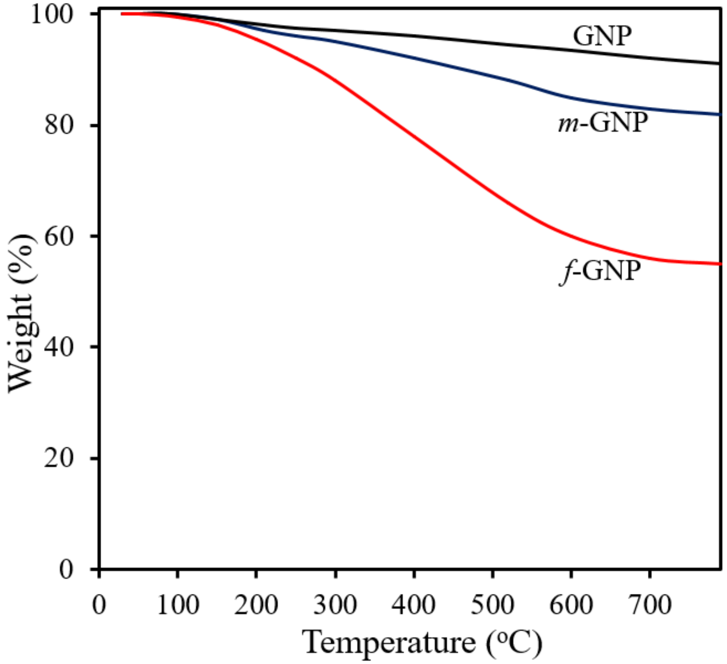

Fig. S1. TGA thermograms of GNP, *m*-GNP and *f*-GNP at heating rate of 10 ^{o}C/min under nitrogen atmosphere.

Table S1. Mechanical properties data of epoxy nanocomposites containing various contents of GNP and *f*-GNP.

		Epoxy/GNP Nanocomposites		*Epoxy/f-GNP Nanocomposites*	
Nanoplatelets (wt %)	Nanoplatelets (vol %)	Tensile Strength (MPa)	Tensile Modulus (GPa)	Tensile Strength (MPa)	Tensile Modulus (GPa)
0	0	58.18±1.7	2.10±0.011	58.18±1.7	2.10±0.011
0.5	0.29	68.1±1.8	2.18±0.017	89.94±2.1	2.28±0.015
1	0.59	56.73±2.5	2.23±0.020	74.65±1.6	2.39±0.026
2	1.19	49.60±2.1	2.43±0.025	67.67±1.9	2.63±0.032
5	3.01	37.84±3.5	2.89±0.034	56.51±3.4	3.27±0.041
		Flexural Strength (MPa)	Flexural Modulus (GPa)	Flexural Strength (MPa)	Flexural Modulus (GPa)
0	0	72.3±2.7	2.40±0.023	72.3±2.7	2.40±0.023
0.5	0.29	88.18± 1.7	2.51±0.019	119.93±2.1	2.78±0.026
1	0.59	69.02± 1.8	2.73±0.033	99.25±2.2	3.11±0.024
2	1.19	58.54± 2.9	2.95±0.041	88.58±3.6	3.29±0.039
5	3.01	50.18± 2.8	3.45±0.038	75.87±3.4	3.95±0.042

Table S2. Summary of the effect of different graphene types and chemical treatments on the tensile, flexural properties, and glass transition temperature in nanocomposites containing low loadings of graphene as reported in the literature.

Graphene type (0.5wt% ,unless otherwise mentioned)	Tensile properties Approximate percentage differences (%) [a]		Flexural properties Approximate percentage differences (%) [a]		T_g (%)[b]	Comments	Ref.
	Modulus	Strength	Modulus	Strength			
GNP with no modification	+3.8	+17	+4.6	+22	+19.3	Better wettability and dispersion/ Strong interfacial adhesion/ Crack deviations/Crack bridging/ Grapehene pull-out resistance/ Mechanical locking	The present work.
Tetraethylenepentamine functionalized GNP	+8.6	+54.6	+15.8	+65.8			
GNP with no modification	+3.2	-4.6	-----		-----	Homogenous dispersion/ Good bonded interface/ Prevention of crack propagation/ Crack barrier effect	9
4,4`-diaminodiphenylsulfone Modified GNP	+53.2	-1.5					
Graphene	+23.86	-6.1	-5.6	-35.9	-----	Homogeneous dispersion/Strong interfacial interaction	10
A bio-based epoxy monomer (GA-II) functionalized graphene	+36	+18.2	+11.5	+7.8			
Ammonia-modified graphene nanosheets	+5.2	+27.8	+7.7	+41.4	+6[c]	Homogeneous dispersion/ Strong interfacial interactions/ Pull-out resistance/ Crack propagation resistance	11
GNP with no modification	+14.6	+14.9	-----		+1.9	Better dispersion/ Crack initiation and propagation resistance/ Efficient stress-transfer/ Crack deflection/ Crack bridging/ Strong interfacial interactions	12
Polybenzimidazole functionalized GNP	+27.15	+26.7					
Synthesized a water-soluble perylene bisimide modified graphene	+81	+36.6	+16.3	+44.2	-----	uniform dispersion/ Strong interfacial interactions	13
2-(4-aminophenyl) ethanol modified graphene (0.4 wt%)[d]	-----		+59.93	+54.1	-4.6[c]	Promoted molecular level of dispersion/ Higher efficiency of load transfer at the interface/ Crack propagation resistance/ Crack deflection	14

<table>
<tr><td colspan="2">Polyoxyalkyleneamine (B200 surfactant) modified GNP</td><td>+17.3</td><td>-19.6</td><td colspan="2">-----</td><td>+8[c]</td><td>Improved dispersion/ Improved filler-matrix interface</td><td>15</td></tr>
<tr><td colspan="2">GNP</td><td colspan="2" rowspan="2">-----</td><td rowspan="2">-----</td><td>-14.5</td><td rowspan="2">-----</td><td rowspan="2">Improvement of interfacial adhesion</td><td rowspan="2">16</td></tr>
<tr><td colspan="2">Brominated GNP</td><td>+9</td></tr>
<tr><td colspan="2">GNP with no modification</td><td>+2.10</td><td>+16</td><td colspan="2" rowspan="4">-----</td><td rowspan="4">-----</td><td rowspan="4">Enhanced dispersion/ Crack deflection/ Crack bridging/ Better GNPs’ pull-out resistance</td><td rowspan="4">17</td></tr>
<tr><td rowspan="3">GNP modification</td><td>oxide</td><td>+6.25</td><td>+36</td></tr>
<tr><td>amine</td><td>+10.4</td><td>+50</td></tr>
<tr><td>silane</td><td>+14</td><td>+40</td></tr>
<tr><td colspan="2">GNP (1 wt%)[d]</td><td colspan="2" rowspan="2">-----</td><td>+3.2</td><td>-11.7</td><td rowspan="2">+1.4</td><td rowspan="2">Uniform dispersion/ Promotion of cross-linking reactions of epoxy and hardener/ Improvement of interfacial binding and the mechanical interactions/ Tilting and twisting of the cracks</td><td rowspan="2">18</td></tr>
<tr><td colspan="2">Amine-terminated poly(butadieneco-acrylonitrile) functionalized GNP (1 wt%)[d]</td><td>+8.4</td><td>-5.8</td></tr>
<tr><td colspan="2">GNP (1 wt%)[d]</td><td colspan="2">-----</td><td>+9</td><td>Not reported</td><td>-----</td><td>Crack deflection and crack bridging</td><td>19</td></tr>
<tr><td colspan="2">Imidazole functionalized reduced GO (0.4 wt%)[d]</td><td>+11.5</td><td>+97.2</td><td colspan="2">-----</td><td>-6.1[c]</td><td>Homogeneous dispersion/ Covalent bonding at the interface</td><td>20</td></tr>
<tr><td colspan="2">GO with no modification</td><td>+6.7</td><td>+22.3</td><td colspan="2" rowspan="2">-----</td><td rowspan="2">+1.5</td><td rowspan="2">Improved compatibility and dispersion/ Strong interfacial interaction/ Crack resistance</td><td rowspan="2">21</td></tr>
<tr><td colspan="2">diglycidyl ether of bisphenol-A functionalized GO</td><td>+16.5</td><td>+61.4</td></tr>
<tr><td colspan="2">GO</td><td>+6.6</td><td>+20</td><td>+4.8</td><td>-10.8</td><td>+0.7</td><td></td><td>22</td></tr>
</table>

3-glycidoxypropyltrimethoxy silane functionalized GO	+14	+37.3	+12.5	-3.8		Enhanced dispersion level/ Strong sheet-matrix bonding/ Crack tip bifurcation/ Crack pinning/ Crack bridging	
GO	+6.7	+22.3	-----		-0.6	Modified dispersion/Chemical interfacial Bonding/ High Energy dissipation	23
Polyetheramine-functionalized GO[e]	+10.8	+57.2					
GO	+24	+11.3	-----		+0.6[c]	Uniform dispersion/ Strong filler-matrix interface/ Deflection of propagating crack fronts	24
GO modification: Base-washed	+16	+7	-----		+0.8		
GO modification: 3-amino propyl trimethoxysilane	+20	+13.2			+0.1		
GO modification: 3-glycidoxy propyl trimethoxysilane	+16	+12.3			-0.7		
Microwave exfoliated reduced GO	+10.9	+5.1	+12.8	+23.4	Without change	Good dispersion/ Good interfacial chemical bonding/ Disturbing and resisting the development of crack growth and prevented crack propagation/Enhanced energy absorption/ Crack bridging	25
Thermal exfoliated graphene (0.2 wt%)[d]	Not reported	+39	+15	+10	+14.8	A stable homogeneous structure in the formation of composites/ Good dispersibility and ordered arrangement/ Improved interface adhesion	26
Poly(amidoamine) modifed GO (3D graphene skeleton) (0.2 wt%)[d]		+141	+75	+43.2			
Thermally reduced graphene (TRG); (0.1 wt%)[d]	-----		+8.7	+15	+1.3	Improved dispersion/ Strong interface/ Mechanical interlocking/ Better Wettability	27

Carboxylic functionalized TRG, (0.1 wt%)[d]			+18.7	+22			
GO	+6.3	+32.7	-----		+3.7	Well dispersion/ Better load transferring from the matrix to the reinforcements/ Induction of high anisotropy in properties via self-aligned along the in-plane direction	28
Highly stable reduction of GO (Using hydrazine)	+15.6	+46.4					
4-aminobenzoyl-functionalized graphite	+23	+8.2	-----		-2.8[c]	Fiber-filler chemical bonding/ Efficient dispersion and uniform distribution	29
triglycidyl para-aminophenol (TGPAP) treated GO EP20-GO1.0[f]	Not reported	+91.8	+37.7	+36.5	-17.5[c]	Superior dispersion and exfoliation/ Strong interfacial interactions/ Crack deflection/ Effective phase transfer agent	30
GO	+8	+13	+11	+23	2.4[c]	Covalent interaction at the graphene-matrix interface/ Reduction in crack growth/ Arrest crack growth/ Crack pinning/ Pullout or bridging mechanism	31
Reduced GO (0.1 wt%)[d]	+31	+40	-----		-----	Frictional pull-out/ Improvement of mechanical interlocking-adhesion at the nanofiller-matrix interface/ Crack deflection	32
Thermally reduced graphite oxide	-18	-7	-----		-----	Mechanical interlocking/ Better adhesion/ Crack deflection	33

Anisotropic graphene aerogel	-----	+12.4	+1.1	-----	Reduction of difficulties to uniformly disperse/ Crack tip blunting and deflection/ Obstructing crack propagation	34
CVD graphene foam	-----	+25.5	+35.1	+35.8[c]	Integrated 3D structure of GF can totally eliminate the issues of uniform dispersion/ Interlocking mechanism/ Enhancing the interfacial adhesion/ Inducing local crack tip blunting/ Formation of microcracking bifurcation dilatation fracture process zone	35

a) Percentage increase "+" or percentage decrease "-" of mechanical properties compared to neat epoxy.
b) Percentage increase "+" or percentage decrease "-" of glass transition temperature compared to untreated nanoparticle/epoxy nanocomposite.
c) The value states the percentage differences of T_g compared with neat epoxy glass transition temperature.
d) The value in brackets indicates that the content of the graphene-based nanofiller which is either lower or higher than 0.5 wt%.
e) An average amount for mechanical properties data are reported for any length of polyether amine coupling agent which is grafted to GO.
f) The prepared epoxy/GO mixtures or nanocomposites are denoted as EPx-GOy, where x is the TGPAP weight percentage in the epoxy oligomer, and y is the GO content in parts per hundred parts of epoxy resin without a curing agent (phr).

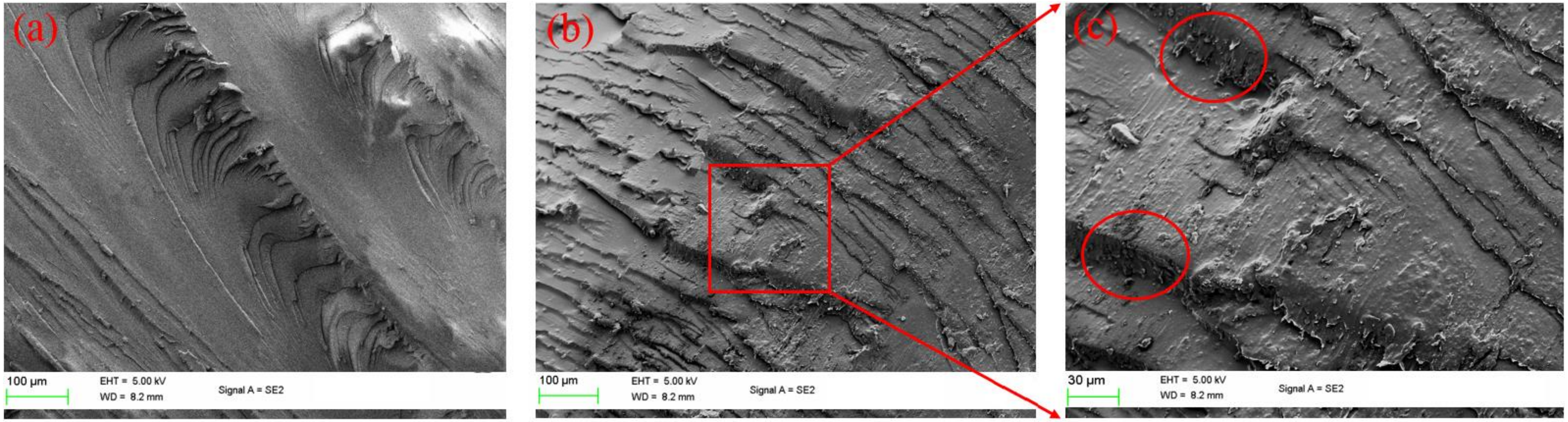

Fig. S2. SEM images of fracture surfaces of pure epoxy (a) and epoxy/0.5% GNP nanocomposites (b & c).

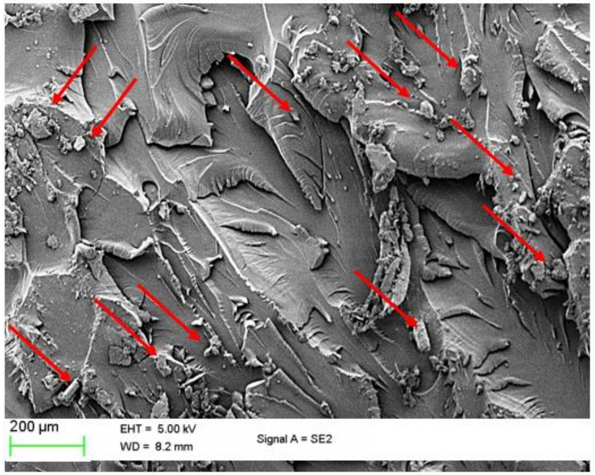

Fig. S3. SEM image of fracture surface of epoxy/5% *f*-GNP nanocomposites containing high level of agglomerations (red arrow).

Reference:

1 Ji, Z., Chen, J., Huang, L. & Shi, G. High-yield production of highly conductive graphene via reversible covalent chemistry. *Chemical Communications* **51**, 2806-2809, doi:10.1039/C4CC09144B (2015).

2 Frolova, L. V. *et al.* Tetracyanoethylene oxide-functionalized graphene and graphite characterized by Raman and Auger spectroscopy. *Carbon* **81**, 216-222, doi:http://dx.doi.org/10.1016/j.carbon.2014.09.052 (2015).

3 Sarkar, S., Bekyarova, E., Niyogi, S. & Haddon, R. C. Diels−Alder Chemistry of Graphite and Graphene: Graphene as Diene and Dienophile. *Journal of the American Chemical Society* **133**, 3324-3327, doi:10.1021/ja200118b (2011).

4 Huang, X. *et al.* Polyhedral Oligosilsesquioxane-Modified Boron Nitride Nanotube Based Epoxy Nanocomposites: An Ideal Dielectric Material with High Thermal Conductivity. *Advanced Functional Materials* **23**, 1824-1831, doi:10.1002/adfm.201201824 (2013).

5 Park, Y. T. *et al.* Epoxy Toughening with Low Graphene Loading. *Advanced Functional Materials* **25**, 575-585, doi:10.1002/adfm.201402553 (2015).

6 Chen, Y. *et al.* High-Performance Epoxy Nanocomposites Reinforced with Three-Dimensional Carbon Nanotube Sponge for Electromagnetic Interference Shielding. *Advanced Functional Materials* **26**, 447-455, doi:10.1002/adfm.201503782 (2016).

7 Zabihi, O., Khayyam, H., Fox, B. L. & Naebe, M. Enhanced thermal stability and lifetime of epoxy nanocomposites using covalently functionalized clay: experimental and modelling. *New Journal of Chemistry* **39**, 2269-2278, doi:10.1039/C4NJ01768D (2015).

8 Negrete-Herrera, N., Putaux, J.-L., David, L. & Bourgeat-Lami, E. Polymer/Laponite Composite Colloids through Emulsion Polymerization: Influence of the Clay Modification Level on Particle Morphology. *Macromolecules* **39**, 9177-9184, doi:10.1021/ma0610515 (2006).

9 Ma, J. *et al.* Covalently bonded interfaces for polymer/graphene composites. *Journal of Materials Chemistry A* **1**, 4255-4264, doi:10.1039/C3TA01277H (2013).

10 Cao, L. *et al.* How a bio-based epoxy monomer enhanced the properties of diglycidyl ether of bisphenol A (DGEBA)/graphene composites. *Journal of Materials Chemistry A* **1**, 5081-5088, doi:10.1039/C3TA01700A (2013).

11 Zhang, D.-D., Zhao, D.-L., Yao, R.-R. & Xie, W.-G. Enhanced mechanical properties of ammonia-modified graphene nanosheets/epoxy nanocomposites. *RSC Advances* **5**, 28098-28104, doi:10.1039/C5RA00312A (2015).

12 Zhang, Y. *et al.* Tuning the interface of graphene platelets/epoxy composites by the covalent grafting of polybenzimidazole. *Polymer* **55**, 4990-5000, doi:http://dx.doi.org/10.1016/j.polymer.2014.07.045 (2014).

13 Pan, L. *et al.* Improving thermal and mechanical properties of epoxy composites by using functionalized graphene. *RSC Advances* **5**, 60596-60607, doi:10.1039/C5RA09410K (2015).

14 Fang, M. *et al.* Constructing hierarchically structured interphases for strong and tough epoxy nanocomposites by amine-rich graphene surfaces. *Journal of Materials Chemistry* **20**, 9635-9643, doi:10.1039/C0JM01620A (2010).

15 Zaman, I. *et al.* A Facile Approach to Chemically Modified Graphene and its Polymer Nanocomposites. *Advanced Functional Materials* **22**, 2735-2743, doi:10.1002/adfm.201103041 (2012).

16 Li, J., Vaisman, L., Marom, G. & Kim, J.-K. Br treated graphite nanoplatelets for improved electrical conductivity of polymer composites. *Carbon* **45**, 744-750, doi:http://dx.doi.org/10.1016/j.carbon.2006.11.031 (2007).

17 Ahmadi-Moghadam, B., Sharafimasooleh, M., Shadlou, S. & Taheri, F. Effect of functionalization of graphene nanoplatelets on the mechanical response of graphene/epoxy composites. *Materials & Design* **66, Part A**, 142-149, doi:http://dx.doi.org/10.1016/j.matdes.2014.10.047 (2015).

18 Wang, F., Drzal, L. T., Qin, Y. & Huang, Z. Effects of functionalized graphene nanoplatelets on the morphology and properties of epoxy resins. *High Performance Polymers*, doi:10.1177/0954008315588983 (2015).

19 Chatterjee, S. *et al.* Size and synergy effects of nanofiller hybrids including graphene nanoplatelets and carbon nanotubes in mechanical properties of epoxy composites. *Carbon* **50**, 5380-5386, doi:http://dx.doi.org/10.1016/j.carbon.2012.07.021 (2012).

20 Liu, W. *et al.* Simultaneous catalyzing and reinforcing effects of imidazole-functionalized graphene in anhydride-cured epoxies. *Journal of Materials Chemistry* **22**, 18395-18402, doi:10.1039/C2JM32708B (2012).

21 Wan, Y.-J. *et al.* Grafting of epoxy chains onto graphene oxide for epoxy composites with improved mechanical and thermal properties. *Carbon* **69**, 467-480, doi:http://dx.doi.org/10.1016/j.carbon.2013.12.050 (2014).

22 Wan, Y.-J., Gong, L.-X., Tang, L.-C., Wu, L.-B. & Jiang, J.-X. Mechanical properties of epoxy composites filled with silane-functionalized graphene oxide. *Composites Part A: Applied Science and Manufacturing* **64**, 79-89, doi:http://dx.doi.org/10.1016/j.compositesa.2014.04.023 (2014).

23 Guan, L.-Z. *et al.* Toward effective and tunable interphases in graphene oxide/epoxy composites by grafting different chain lengths of polyetheramine onto graphene oxide. *Journal of Materials Chemistry A* **2**, 15058-15069, doi:10.1039/C4TA02429J (2014).

24 Li, Z. *et al.* Control of the functionality of graphene oxide for its application in epoxy nanocomposites. *Polymer* **54**, 6437-6446, doi:http://dx.doi.org/10.1016/j.polymer.2013.09.054 (2013).

25 T.K, B. S., Nair, A. B., Abraham, B. T., Beegum, P. M. S. & Thachil, E. T. Microwave exfoliated reduced graphene oxide epoxy nanocomposites for high performance applications. *Polymer* **55**, 3614-3627, doi:http://dx.doi.org/10.1016/j.polymer.2014.05.032 (2014).

26 Ni, Y. *et al.* Superior Mechanical Properties of Epoxy Composites Reinforced by 3D Interconnected Graphene Skeleton. *ACS Applied Materials & Interfaces* **7**, 11583-11591, doi:10.1021/acsami.5b02552 (2015).

27 Naebe, M. *et al.* Mechanical Property and Structure of Covalent Functionalised Graphene/Epoxy Nanocomposites. *Scientific Reports* **4**, 4375, doi:10.1038/srep04375 (2014).

28 Yousefi, N. *et al.* Simultaneous in situ reduction, self-alignment and covalent bonding in graphene oxide/epoxy composites. *Carbon* **59**, 406-417, doi:http://dx.doi.org/10.1016/j.carbon.2013.03.034 (2013).

29 Kim, K.-S., Jeon, I.-Y., Ahn, S.-N., Kwon, Y.-D. & Baek, J.-B. Edge-functionalized graphene-like platelets as a co-curing agent and a nanoscale additive to epoxy resin. *Journal of Materials Chemistry* **21**, 7337-7342, doi:10.1039/C0JM03504A (2011).

30 Tang, X., Zhou, Y. & Peng, M. Green Preparation of Epoxy/Graphene Oxide Nanocomposites Using a Glycidylamine Epoxy Resin as the Surface Modifier and Phase Transfer Agent of Graphene Oxide. *ACS Applied Materials & Interfaces* **8**, 1854-1866, doi:10.1021/acsami.5b09830 (2016).

31 Bortz, D. R., Heras, E. G. & Martin-Gullon, I. Impressive Fatigue Life and Fracture Toughness Improvements in Graphene Oxide/Epoxy Composites. *Macromolecules* **45**, 238-245, doi:10.1021/ma201563k (2012).

32 Rafiee, M. A. *et al.* Enhanced Mechanical Properties of Nanocomposites at Low Graphene Content. *ACS Nano* **3**, 3884-3890, doi:10.1021/nn9010472 (2009).

33 Rafiee, M. A. *et al.* Fracture and fatigue in graphene nanocomposites. *small* **6**, 179-183 (2010).

34 Wang, Z. *et al.* Graphene Aerogel/Epoxy Composites with Exceptional Anisotropic Structure and Properties. *ACS Applied Materials & Interfaces* **7**, 5538-5549, doi:10.1021/acsami.5b00146 (2015).

35 Jia, J. *et al.* Exceptional Electrical Conductivity and Fracture Resistance of 3D Interconnected Graphene Foam/Epoxy Composites. *ACS Nano* **8**, 5774-5783, doi:10.1021/nn500590g (2014).
